# Swept away: ocean currents and seascape features influence genetic structure across the 18,000 Km Indo-Pacific distribution of a marine invertebrate, the black-lip pearl oyster *Pinctada margaritifera*

**DOI:** 10.1186/s12864-016-3410-y

**Published:** 2017-01-10

**Authors:** Monal M. Lal, Paul C. Southgate, Dean R. Jerry, Cyprien Bosserelle, Kyall R. Zenger

**Affiliations:** 1 Centre for Sustainable Tropical Fisheries and Aquaculture, and College of Science and Engineering, James Cook University, Townsville, QLD 4811 QLD Australia; 2 Australian Centre for Pacific Islands Research, Faculty of Science, Health, Education and Engineering, University of the Sunshine Coast, Maroochydore, QLD 4558 QLD Australia; 3 Geoscience Division, Secretariat of the Pacific Community, 241 Mead Road, Nabua, Suva Fiji Islands

**Keywords:** Population genomics, Aquaculture, Core-periphery hypothesis, SNP, Hydrodynamic dispersal, Species distribution

## Abstract

**Background:**

Genetic structure in many widely-distributed broadcast spawning marine invertebrates remains poorly understood, posing substantial challenges for their fishery management, conservation and aquaculture. Under the Core-Periphery Hypothesis (CPH), genetic diversity is expected to be highest at the centre of a species’ distribution, progressively decreasing with increased differentiation towards outer range limits, as populations become increasingly isolated, fragmented and locally adapted. The unique life history characteristics of many marine invertebrates such as high dispersal rates, stochastic survival and variable recruitment are also likely to influence how populations are organised. To examine the microevolutionary forces influencing population structure, connectivity and adaptive variation in a highly-dispersive bivalve, populations of the black-lip pearl oyster *Pinctada margaritifera* were examined across its ~18,000 km Indo-Pacific distribution.

**Results:**

Analyses utilising 9,624 genome-wide SNPs and 580 oysters, discovered differing patterns of significant and substantial broad-scale genetic structure between the Indian and Pacific Ocean basins. Indian Ocean populations were markedly divergent (*F*_st_ = 0.2534–0.4177, *p* < 0.001), compared to Pacific Ocean oysters, where basin-wide gene flow was much higher (*F*_st_ = 0.0007–0.1090, *p* < 0.001). Partitioning of genetic diversity (hierarchical AMOVA) attributed 18.1% of variance between ocean basins, whereas greater proportions were resolved within samples and populations (45.8% and 35.7% respectively). Visualisation of population structure at selectively neutral loci resolved three and five discrete genetic clusters for the Indian and Pacific Oceans respectively. Evaluation of genetic structure at adaptive loci for Pacific populations (89 SNPs under directional selection; *F*_st_ = 0.1012–0.4371, FDR = 0.05), revealed five clusters identical to those detected at neutral SNPs, suggesting environmental heterogeneity within the Pacific. Patterns of structure and connectivity were supported by Mantel tests of isolation by distance (IBD) and independent hydrodynamic particle dispersal simulations.

**Conclusions:**

It is evident that genetic structure and connectivity across the natural range of *P. margaritifera* is highly complex, and produced by the interaction of ocean currents, IBD and seascape features at a broad scale, together with habitat geomorphology and local adaptation at regional levels. Overall population organisation is far more elaborate than generalised CPH predictions, however valuable insights for regional fishery management, and a greater understanding of range-wide genetic structure in a highly-dispersive marine invertebrate have been gained.

**Electronic supplementary material:**

The online version of this article (doi:10.1186/s12864-016-3410-y) contains supplementary material, which is available to authorized users.

## Background

Understanding the patterns and processes shaping population genetic structure across the extent of a species’ distribution is an important prerequisite for biological conservation and management efforts, as well as studies of speciation [1]. For marine taxa, regional fishery management and aquaculture practices also rely on biologically meaningful population structure to delineate discrete stocks [2–4]. The ability to quantify genetic variation across the geographical limits of a species may shed light on why species might demonstrate stable range boundaries, and also permit assessment of the conservation value of central (*C*) versus marginal (*M*) populations [1, 5, 6]. Several studies (reviewed by Eckert et al. [5] and Sexton et al. [6]), have investigated the central-marginal (*C-M*) hypothesis, also known as the core-periphery hypothesis (CPH; [5, 7, 8]). While many comparisons between taxa have revealed a general decline in genetic diversity and increased differentiation towards range margins, others show no clear patterns [1].

It is expected that the interplay of microevolutionary forces, (namely natural selection, genetic drift and gene flow), will largely determine the magnitude and extent of population structure and connectivity, although the spatial distribution and demographic characteristics of the species could also exert strong influences [5, 6]. The CPH provides a model for interpreting how microevolutionary forces may shape genetic divergence patterns throughout a species’ range. Under this model, a species which colonises a geographical gradient of environmental conditions, is over time expected to exhibit maximised abundance (highest survival, reproduction and growth rates) around a central point where conditions are optimal, while populations become smaller, more fragmented, increasingly divergent and influenced by selective forces towards the periphery [5, 7, 9]. However, exactly how the abundant centre distribution relates to the partitioning of genetic diversity, patterns of differentiation and adaptive differences across the *C-M* cline, remains a contentious topic [5, 9]. One explanation offered suggests that both effective population size (*N*_e_) and gene flow (*m*) should be highest at the centre, and lowest at range margins. Consequently, central populations are expected to be less genetically differentiated and possess higher levels of genetic diversity, than those existing at range margins [5, 7]. Furthermore, due to environmental heterogeneity across a *C-M* cline, local adaptation may be observed between populations existing at the core and range peripheries.

While several studies have examined *C-M* genetic patterns in terrestrial taxa [5, 10], comparatively few investigations have involved marine species [8], and marine invertebrates in particular [11]. Marine systems present several challenges for range-wide studies, as >70% of invertebrates and many vertebrates are characterised by large population sizes, high fecundity, external fertilisation and larvae that typically remain in the plankton for several weeks, although this may vary anywhere from a few minutes to years [12–16]. Consequently, *C-M* patterns compared to terrestrial taxa may differ from expectations under the CPH, as the homogenising influence of gene flow may maintain high connectivity across the *C-M* cline [8]. Furthermore, divergence and local adaptation may not be as apparent if populations remain highly connected, and environmental gradients are shallow.

Among marine invertebrates, species which are either completely sessile as adults (e.g. barnacles, sponges and ascidians), or possess very limited mobility (e.g. sea urchins, bivalves, gastropods), present additional challenges for assessment of *C-M* trends [17, 18]. As larvae undergo pelagic dispersal and recruitment, differential selective pressures and survival rates pre- and post-settlement, and also between the plankton and benthos may strongly influence the genetic composition of populations [19, 20]. Furthermore, the spatial distribution of a population may be limited to isolated biodiversity hotspots (e.g. single bivalve beds), or an entire reef shelf [21, 22].

Given the complex nature of the biological and environmental influences at play, it is important to consider multiple sources of information for range-wide investigations in the marine environment, particularly when the species being examined is extensively distributed across heterogenous habitats. Considerations that have been highlighted in previous analyses of *C-M* patterns involving terrestrial taxa, include examination of the geographical direction of the periphery studied, latitudinal effects, the effects of species-range geometry (e.g. shape and size), as well as sampling strategy [1, 5, 10]. While not all of these may apply to marine scenarios, for taxa that employ a broadcast spawning reproductive strategy, consideration of the extent of ocean current-mediated larval dispersal addresses many of these points [4, 23–26].

Incorporation of environmental data such as dispersal modelling into range-wide studies is capable of offering unprecedented insights into larval dispersal limits [4, 25, 27–29], and when considered together with both neutral and adaptive patterns of population structure, permit a holistic assessment of concordance with the CPH, or other models of range-wide structuring. The advantage of using independent datasets also includes the potential to reveal and/or corroborate previously undiscovered or poorly understood biogeographic barriers to dispersal, cryptic speciation and regional local adaptation [30–33].

**Fig. 1:**
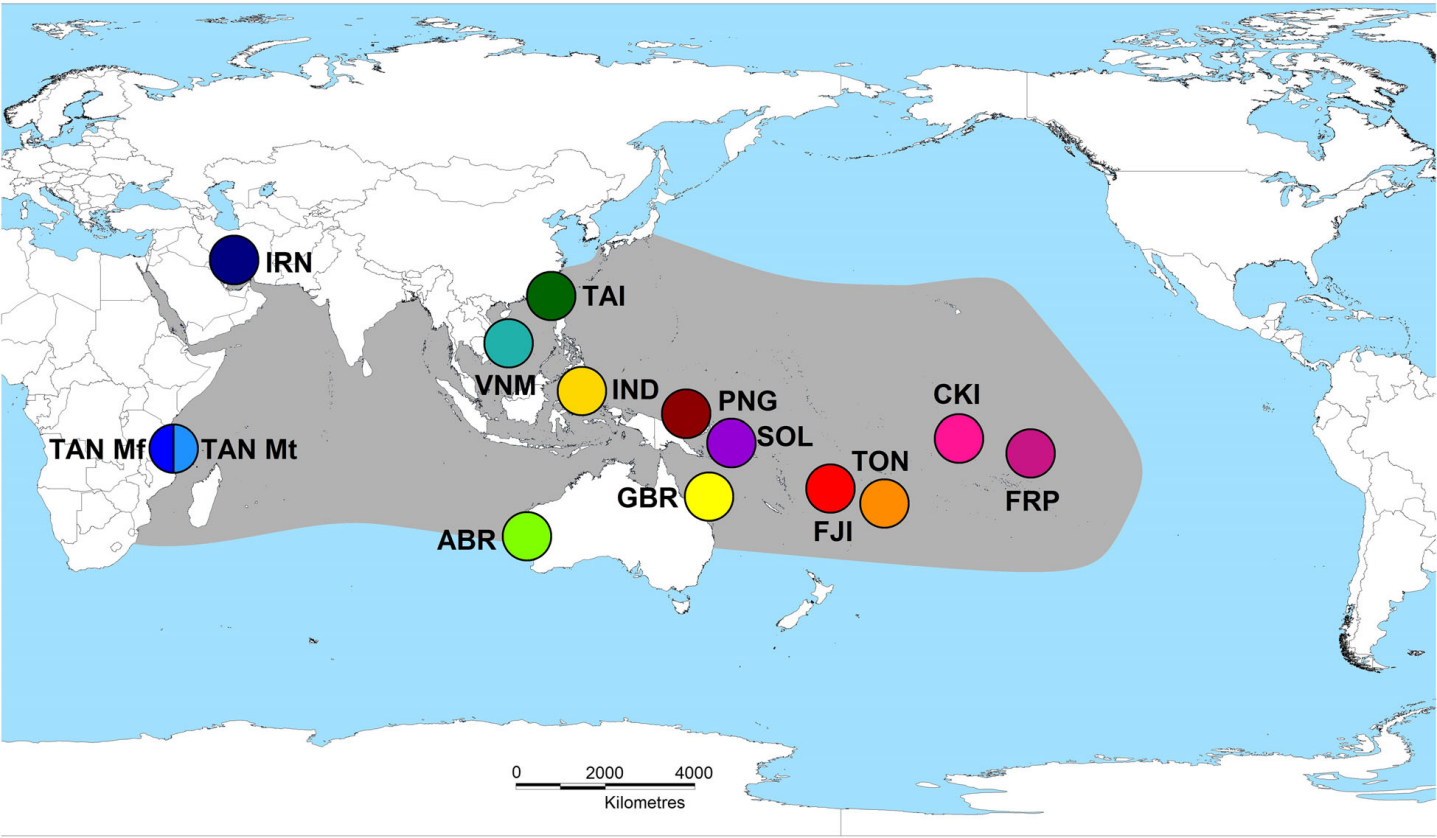
Map of global sampling locations from where 580 individuals of *P. margaritifera* were collected. The approximate known distribution and range of the species is presented in grey, and adapted from Wada and Tëmkin [35]. Site codes represent the following locations: TAN Mf: Mafia Island, Tanzania (dark blue); TAN Mt: Mtwara, Tanzania (light blue); IRN: Hendorabi Island, Iran; TAI: Checheng, Taiwan; VNM: Nha Trang, Vietnam; IND: Manado, Indonesia; AU Abr: Abrolhos Islands, Australia; AU GBR: Great Barrier Reef, Australia; PNG: Kavieng, Papua New Guinea; SOL: Gizo Island, Solomon Islands; FJI: Kadavu, Savusavu, Lau and the Yasawa group, Fiji Islands; TON: Tongatapu, Tonga; CKI: Manihiki Atoll, Cook Islands and FRP: Arutua, French Polynesia

The black-lip pearl oyster *Pinctada margaritifera* (Pteriidae), is a marine bivalve mollusc that has a broad Indo-Pacific distribution (Fig. 1), and is highly valued for cultured pearl and pearl shell production [34, 35]. Aquaculture of this species comprises a valuable industry and important source of coastal community livelihood across almost the entire extent of its distribution [34, 36]. While analyses to examine population structure and connectivity have previously been carried out, these have produced mixed findings, incorporated a range of different marker types (allozymes, mtDNA and microsatellites), and never examined the entirety of the species distribution [19, 37–43]. The current species description includes a total of six sub-species [35, 44, 45], that are described exclusively on the basis of variable morphological characters [46]. In the Pacific basin, Hawaiian populations are known as *P. margaritifera* var. *galstoffi* (Bartsch, 1931), Cook Islands and French Polynesian individuals as *P. m.* var. *cummingi* (Reeve, 1857), and all Central and Western Pacific specimens as *P. m.* var. *typica* (Linnaeus, 1758). Indian Ocean populations are represented by *P. m.* var. *persica* (Jameson, 1901; Persian Gulf), *P. m.* var. *erythraensis* (Jameson, 1901; Red Sea) and *P. m.* var. *zanzibarensis* (Jameson, 1901; East Africa, Madagascar and Seychelle Islands [44]).

Significant genetic heterogeneity has been reported for *P. margaritifera* at nuclear markers (allozymes, anDNA markers and microsatellite loci), at various sites in the Western and Central Pacific [37, 42, 47], while contrastingly mitochondrial markers did not [37]. More recent work also using microsatellite loci, discovered significant genetic structure both within and between French Polynesian island archipelagos, attributed to “open” and “closed” atoll lagoon hydromorphologies restricting patterns of gene flow [39]. Since then, genome-wide SNPs have been developed and characterised [48], and used to investigate stock structure for fishery management and aquaculture in the Fiji Islands [4], where a single genetic stock was identified.

Previous studies of range-wide genetic structuring in Pteriid pearl oysters have produced mixed results. Lind et al. [49] reported a reduction in genetic diversity towards the range periphery of the silver-lip pearl oyster, *P. maxima*, which is consistent with CPH assumptions. However, the natural distribution of this species is considerably less extensive than that of *P. margaritifera* [34, 35]. A bivalve which has a range similar to that of *P. margaritifera* is the Akoya pearl oyster, currently recognised as the *P. fucata*/*martensii*/*radiata*/*imbricata* species complex [35, 50]. While the population genetic structure of this taxon is pending resolution, it is thought that it may comprise one cosmopolitan, circum-globally distributed species, possessing a very high degree of intraspecific variation across its range [34, 35, 51].

Larval development of *P. margaritifera* occurs over 26–30 days in captivity [52, 53], however, time to settlement may be prolonged if conditions are unfavourable [54]. The high dispersal potential (and thus gene flow) in this species suggests that CPH trends may not be easily identifiable across the broader species range, except perhaps in situations where larval dispersal is restricted by seascape features (e.g. closed atoll lagoons or current gyres), or at the very limits of the species distribution where favourable habitat is limited, impacting fitness and population growth. Here, we assess populations of *P. margaritifera* across the extent of its Indo-Pacific distribution spanning over 18,000 km, and compare our observations with expectations under the CPH and regional morphological subdivisions. Independent population genomic and hydrodynamic approaches were utilised to assess population genetic structure, adaptive variation and larval connectivity. Through the use of independent biological and environmental datasets, this work sheds light on the links between genetic structure, ecology and oceanography, to reveal how populations of a broadcast spawner can be organised and maintained in the marine environment.

## Methods

### Specimen collection, tissue sampling and DNA extraction

Adult and juvenile *P. margaritifera* (*n* = 580) between 5 and 18 cm in dorso-ventral measurement (DVM) were collected from 14 sites across the species distribution (Fig. 1). All oysters were handled in accordance with James Cook University’s animal ethics requirements and guidelines, with permission to collect tissues obtained from local authorities. In the Indian Ocean, oysters were collected from two sites in Tanzania (Mafia Island and Mtwara, *n* = 35 and *n* = 20 respectively), the Persian Gulf (Hendorabi Island, Iran; *n* = 49) and Post Office Island in the Abrolhos Islands group, Western Australia (*n* = 50). All Indian Ocean samples consisted of wild individuals with the exception of the Abrolhos Islands collection, where oysters were hatchery-produced from wild-caught broodstock. In the Western Pacific, oysters were sampled from Checheng, Taiwan (*n* = 24), Nha Trang, Vietnam (*n* = 47) and Manado, Indonesia (*n* = 48). Central Pacific locations were represented by Kavieng, Papua New Guinea (*n* = 38), Gizo Island in the Solomon Islands (*n* = 50), the Great Barrier Reef, Australia (*n* = 35), the Fiji Islands (*n* = 61) and Tonga (*n* = 28). In the Eastern Pacific, oysters were collected from Manihiki Atoll in the Cook Islands (*n* = 45), and Arutua, French Polynesia (*n* = 50). All Pacific Ocean samples consisted of wild oysters, with the exception of the Cook Islands and French Polynesian samples that were sourced from pearl farm stocks.

Proximal mantle and adductor muscle tissues (3 and 6 cm, respectively) were removed and transferred to tubes containing 20% salt saturated dimethyl sulfoxide (DMSO-salt) preservative [55]. Genomic DNA was extracted using a modified cetyl trimethyl ammonium bromide (CTAB, Amresco, cat. #0833-500G) chloroform/isoamyl alcohol protocol with a warm (30 °C) isopropanol precipitation [56]. To clean up all DNA extractions, a Sephadex G50 [57] spin column protocol was used prior to quantification with a Nanodrop 1000 Spectrophotometer (Thermo Scientific). All samples were subsequently normalised at 100 ng/μL in a 50 μL final volume, and submitted for DArTseq™ 1.0 genotyping at Diversity Arrays Technology PL, Canberra, ACT, Australia.

### DArTseq™ 1.0 library preparation and sequencing

Diversity Arrays Technology (DArT PL) proprietary genotyping by sequencing (DArTseq™) reduced-representation libraries were prepared as described by Kilian et al. [58] and Sansaloni et al. [59], with a number of modifications for *P. margaritifera*. Briefly, genome complexity reduction was achieved with a double restriction digest, using a *Pst*I and *Sph*I methylation-sensitive restriction enzyme (RE) combination, in a joint digestion-ligation reaction at 37 °C for 2 h with 150–200 ng gDNA. Because *P. margaritifera* like other bivalve species is highly polymorphic [48, 60], highly repetitive genomic regions were avoided and low copy regions more efficiently targeted for sequence capture with the use of methylation-sensitive REs [61].

Custom proprietary barcoded adapters (6–9 bp) were ligated to RE cut-site overhangs as per Kilian et al. [58], with the adapters designed to modify RE cut sites following ligation, to prevent insert fragment re-digestion. The *Pst*I-compatible (forward) adapter incorporated an Illumina flowcell attachment region, sequencing primer sequence and a varying length barcode region [58, 62]. The reverse adapter also contained a flowcell attachment region, and was compatible with the *Sph*I cut-site overhang. Samples were processed in batches of 94, with 15% of all samples in a batch randomly selected for replication, to provide a basis for assessing region recovery and genotyping reproducibility. Target “mixed” fragments [62], containing both *Sph*I and *Nla*III cut-sites were selectively amplified using custom designed primers for each sample, under the following PCR conditions: initial denaturation at 94 °C for 1 min, then 30 cycles of 94 °C for 20s, 58 °C for 30s and 72 °C for 45 s, followed by a final extension step at 72 °C for 7 min. Amplified samples were subsequently cleaned using a GenElute PCR Clean-up Kit (Sigma-Aldrich, cat.# NA1020-1KT), on a TECAN Freedom EVO150 automated liquid handler.

To examine fragment size concordance and digestion efficiency, all samples were visualised on a 0.8% agarose gel stained with EtBr, and quantified using the ImageJ software package [63]. Samples which did not appear to have undergone complete digestion and/or amplification were removed from downstream library preparation. A total of 580 samples were each normalised and pooled using an automated liquid handler (TECAN, Freedom EVO150), at equimolar ratios for sequencing on the Illumina HiSeq 2500 platform. After cluster generation and amplification (HiSeq SR Cluster Kit V4 cBOT, cat.# GD-401-4001), 77 bp single-end sequencing was performed at the DArT PL facility in Canberra, Australia.

### Sequence quality control, marker filtering and genotype calling at DArT PL

Raw reads obtained following sequencing were processed using Illumina CASAVA v.1.8.2 software for initial assessment of read quality, sequence representation and generation of FASTQ files. Filtered FASTQ files were then supplied to the DArT PL proprietary software pipeline DArTtoolbox, which performed further filtering, variant calling and generated final genotypes in sequential primary and secondary workflows [64]. Within DArTtoolbox, the primary workflow first involved the package DArTsoft14 to remove reads with a quality score <25 from further processing, and apply stringent filtering to the barcode region of all sequences to increase confidence in genomic region recovery. Individual samples were then de-multiplexed by barcode, and subsequently aligned and matched to catalogued sequences in both NCBI GenBank and DArTdb custom databases to check for viral and bacterial contamination, with any matches removed from further processing.

The secondary workflow employed the DArTsoft14 and KD Compute packages along with the DArTdb database, to identify polymorphisms by aligning identical reads to create clusters across all individuals sequenced. These clusters were then catalogued in DArTdb, and matched against each other to create reduced-representation loci (RRL), based on their degree of similarity and size. SNP and reference allele loci were identified within clusters and assigned the following DArT scores: “0” = reference allele homozygote, “1” = SNP allele homozygote and “2” = heterozygote, based on their frequency of occurrence. To ensure robust variant calling, all monomorphic clusters were removed, SNP loci had to be present in both allelic states (homozygous and heterozygous), and a genetic similarity matrix was produced using the first 10,000 SNPs called to assess technical replication error [65], and exclude clusters containing tri-allelic or aberrant SNPs and overrepresented sequences.

Once SNP markers had been confidently identified, each locus was assessed in the KD Compute package for homozygote and heterozygote call rate, frequency, polymorphic information content (PIC), average SNP count, read depth and repeatability, before final genotype scores were supplied by DArT PL. Following the receipt of genotype data from DArT PL, the dataset was further filtered to retain only a single, highly informative SNP at each genomic locus. This was achieved by filtering out duplicate SNPs (possessing identical Clone IDs), according to call rate and Minor Allele Frequency (MAF). Subsequently, loci were screened for call rate, average Polymorphic Information Content (PIC), MAF and average repeatability, to retain SNPs suitable for population genomic analyses. All loci were then tested for departure from Hardy-Weinberg Equilibrium (HWE) using Arlequin v.3.5.1.3 [66], using an exact test with 10,000 steps in the Markov Chain and 100,000 dememorisations. Additionally, all loci were tested for genotypic linkage disequilibrium (LD) in Genepop v.4.3 [67], as per Lal et al. [48]. Two separate datasets were then created, one which contained selectively neutral loci, and the other which included loci putatively under selection. Bayescan v.2.1 and LOSITAN software were used to detect loci under selection, and further details are provided under that section of the methods.

### Evaluation of genomic diversity, inbreeding and population differentiation

For assessment of genomic diversity within and between populations, allelic diversity indices including average observed (*H*_o_) and average expected heterozygosities corrected for population sample size (*H*_n.b._) were computed. Inbreeding coefficient (*F*_is_) calculations and estimation of effective population size based on the linkage disequilibrium method (*N*_*eLD*_), were also carried out for each population, all using Genetix v.4.05.2 [68] and NeEstimator v.2.01 [69]. Average homozygosity by locus (HL), standardised heterozygosity (SH) and internal relatedness (IR) were also computed per individual, with the *R* package *Rhh* [70]. In addition, the average multi-locus heterozygosity (Av. MLH) per population was determined after Slate et al. [71], along with the mean number of alleles per locus (*A*) using the *diveRsity* [72] *R* package. The number of private alleles (*A*_p_) was computed using HP-RARE v.1.0 [73], according to population groups identified from Netview P and DAPC analyses (see results), due to the levels of genetic divergence observed. Furthermore, rare allelic richness (*Ar,* <5% MAF) was computed manually for each population.

### Resolution of broad and fine-scale population structure and connectivity

Pairwise *F*_st_ estimates for each population were calculated using Arlequin v.3.5.1.3 with 10,000 permutations [66], along with a hierarchical Analysis of Molecular Variance (AMOVA) in the *R* package *Poppr* [74]. The AMOVA examined variation between individuals, populations and regions (Pacific vs. Indian Ocean basins). To assess an isolation by distance (IBD) model of gene flow among populations, Mantel tests were carried out using GenAlEx v.6.5 [75], based on pairwise *F*_st_ and straight-line geographic distance matrices over 10,000 permutations. Mantel tests were performed considering populations within each ocean basin together, separately, and also within Pacific Ocean population clusters identified by DAPC and NetView P analyses. Nei’s (1978) standard genetic distances (*D*_S_) between populations were also computed in Genetix v.4.05.2 with 10,000 permutations [68], and broad-scale population structure visualised by performing a Discriminant Analysis of Principal Components (DAPC) in the *R* package *adegenet* 1.4.2 [76–78]. The DAPC was carried out for all loci, and α-score optimisation used to determine the number of principal components to retain. To reveal any fine-scale stratification between and among all populations, network analysis was carried out using the NetView P pipeline v.0.4.2.5 [79, 80]. To further investigate the direction and magnitude of migration between populations, migration networks were generated using the *divMigrate* function of the *R* package *diveRsity*, utilising the Nei’s *G*_st_ method [72, 81].

### Examination of adaptive variation

To first create a selectively neutral dataset for population genomic analyses, a filtered dataset containing 10,683 SNP loci was used as the starting point for this step. Both BayeScan v.2.1 [82, 83] and LOSITAN selection detection workbench [84] software packages were employed to identify candidate loci under selection, at FDRs = 0.001, 0.005, 0.01, 0.05 and 0.1 and 0.2. The numbers of loci detected are summarised in Additional file 3, and verification of these loci was carried out using QQ plots (data not shown). The intended approach was to select loci jointly identified by both Bayescan 2.1 and LOSITAN, at the appropriate FDR threshold determined by QQ plot distribution. As these software packages employ different analytical approaches, their joint use generally increases the statistical confidence of *F*_st_ outlier detection [85–87]. Candidate loci identified with high probability using both methods were to be considered as true outliers, and representative of putative selection impacting the populations examined. However, given the tendency of LOSITAN to overestimate the numbers of loci under selection [32, 48, 88], and disagreement on an appropriate FDR threshold to apply using both methods, a conservative approach was taken where LOSITAN results were disregarded, and the Bayescan 2.1 results at an FDR = 0.01 considered. This indicated that a total of 1,059 putatively balancing and directional loci were present in the dataset, and following their removal, a selectively neutral dataset containing 9,624 SNPs remained.

Further population-specific *F*_*st*_ outlier tests were used to detect local adaptation, with population pairs tested at FDRs of 0.001, 0.005, 0.01, 0.05 and 0.1 and 0.2. However, testing for *F*_*st*_ outliers was restricted to populations sampled from the Pacific Ocean basin, as they were the least differentiated amongst themselves (i.e. lowest neutral *F*_*st*_ levels <0.11; see results), while all Indian Ocean populations were significantly more divergent. Comprehensive descriptions of the settings used for both software packages were as per Lal et al. [4, 48]. Results of the Bayescan 2.1 and LOSITAN analyses, together with the construction of pairs of Quantile-Quantile plots (QQ-plots), were used to assess the suitability of an FDR threshold for outlier detection between the two methods. The *R* package *GWASTools* v.1.14.0 [89] was used to construct all QQ-plots at all FDR levels examined. All loci were included in the first QQ plot constructed to visualise deviation outside the bounds of a 95% confidence interval. If deviation was observed, a second plot was generated excluding all outlier loci. If all remaining loci were normally distributed, this was interpreted as confirmation that outlier loci had been identified with high probability.

### Particle dispersal simulation

To independently evaluate larval connectivity using oceanographic data for comparison with population genomic analyses, larval transport pathways between sampling locations were simulated using the particle dispersal modelling software DisperGPU (https://github.com/CyprienBosserelle/DisperGPU). Larvae of *P. margaritifera* remain in the plankton for 26–30 days prior to settlement [52, 53], and due to very limited motility, are largely dispersed by current advection and turbulent diffusion in the ocean surface (mixed) layer.

### Hydrodynamic and dispersal numerical models

The particle dispersal model was driven by current velocity output from the global HYbrid Coordinate Ocean Model (HYCOM) data [90, 91]. HYCOM is a global hydrodynamic model that simulates ocean surface heights, currents, salinity and temperature, both at the surface and at depth. The model is driven by meteorological forcing, and constantly constrained by the assimilation of global, remote and in-situ ocean observations. As the model simulates regional and global circulation, it does not include tidal or surface wind waves. HYCOM is highly useful for forecasting and simulation experiments, with public availability at https://hycom.org. The HYCOM model had a resolution of 1/12th of a degree and output every day. The particle model used a standard Lagrangian formulation [92, 93], where particles have no physical representation, but rather track the displacement of neutrally buoyant small objects such as larvae (relative to the model resolution), at the ocean surface. Particle displacement is expressed as:

1 $$ \Delta x={u}_p*\Delta t+K $$

Here *x* represents particle position (latitude and longitude), Δ*x* is particle displacement during a time step Δ*t* (which was set at 1 h), and *u*_*p*_ is the surface current speed at the location of the particle. *K* is the eddy diffusivity which takes account of the random displacement of the particle, due to turbulent eddies at a scale smaller than the hydrodynamics model resolution. *K* is calculated after Viikmäe et al. [94] as follows:

2 $$ K=\sqrt{-4{E}_h\Delta t \log \left(1-{R}_{NA}\right)} \cos \left(2\pi {R}_{NB}\right) $$

Here *E*_*h*_ is a horizontal turbulent diffusion coefficient, and *R*_*NA*_ with *R*_*NB*_ are normally distributed random numbers. The horizontal turbulent diffusion coefficient is unknown, but assumed to be 5 m^2^s^−1^ [94] and *u*_*p*_ (in Eq. 1) is calculated by interpolating the velocity from the hydrodynamic model, both spatially and temporally. Gridded surface currents are first interpolated to the dispersal step, after which the current velocity at each particle position is calculated using a bi-linear interpolation of the gridded surface currents, where only surface currents are taken into account and vertical movements neglected [95]. The particle age is retained and increases with simulation progression.

### Model configuration

Particles were seeded in 11 locations corresponding to locations from where oysters were sampled for genetic analyses (see Fig. 5), which were represented at scales larger than the precise sampling locations to factor in the extent of surrounding coral reef habitat, as per Lal et al. [4]. All seed areas were also extended farther offshore to account for the fact that the HYCOM model is not adapted for shallow water environments, and does not resolve fine-scale hydrodynamic patterns <10 km [96]. Dispersal simulations for the Tanzanian and Iranian sites were not explored, due to the considerable distances between locations, and preliminary examination of circulation patterns that predicted a lack of particle admixture.

Within the Pacific basin, *P. margaritifera* is known to have two reproductive events per year, with peaks and duration of spawning events varying by location. In the Indian Ocean, spawning appears to be restricted to a single season [97]. A summary of the number and duration of spawning seasons for each sampling location was compiled from literature, to replicate larval supply over the year (see Additional file 1). At each seed location, 25,600 particles (see Lal et al., [4]) were released per day for 14 days, corresponding to documented spawning peaks for the species, and the model run forward in time for 90 and 60 days for the first and second spawning periods respectively, within a single calendar year. Simulations were run separately for each of the two spawning periods using HYCOM data for 2015 and 2014, which were selected as these corresponded to an El Niño Southern Oscillation event (ENSO), [98, 99]. This permitted evaluation of any changes in dispersal patterns due to ENSO events over the 2014–2015 time scale.

Particle positions were extracted at time intervals of 60 and 90 days post-seeding for the first and second spawning seasons respectively, per year, and particle displacement visualised using the Generic Mapping Tools package [100]. Explicit, quantitative correlation of the genetic and hydrodynamic analyses was not possible, as this would have required genetic analysis of oysters at all potential source and sink locations with dense sampling coverage, and modelling of substantially more complex particle competency behaviour than computational resources permitted. Instead, an independent approach was adopted here, to examine congruency of results produced by the two analyses. No mortality or competency behaviour of the particles was simulated.

## Results

### SNP filtering

The raw dataset contained a total of 19,666 SNPs genotyped across all 580 individuals, at call rates ranging from 20 to 100%. The first filtering step undertaken to remove duplicate (clone) SNPs at genomic loci resulted in the removal of 8,079 SNPs (41% loss), after which the dataset was filtered for call rate (65%), average PIC (1%), MAF (2%) and average repeatability (95%). A total of 7 loci were found to deviate from HWE (*p* < 0.009), and 99 loci were monomorphic across all 14 populations, which were subsequently removed together with 107 loci under significant LD (*p* < 0.0001). These steps collectively resulted in the retention of 10,683 SNPs (Additional file 6). Testing of this filtered dataset for *F*_st_ outlier loci detected 1,059 SNPs determined to be putatively under balancing and directional selection (Bayescan 2.1 results at FDR = 0.01; Additional file 3), and their removal generated a final neutral dataset of 9,624 SNPs (Additional file 5). This dataset was used for performing all population genomic analyses, while the original filtered dataset (10,683 SNPs) was retained for investigating adaptive variation.

### Population genomic diversity and differentiation

Patterns observed in the mean numbers of alleles per locus (*A*) and rare allelic richness (*Ar*, <5% MAF) were similar, and appeared to vary by Ocean basin (Table 1). Values of *A* for Pacific Ocean populations ranged from 1.6256 (Cook Islands) to 1.8067 (Indonesia), whereas Indian Ocean populations produced values of 1.3934–1.5649 (Tanzania, Mtwara to Abrolhos Islands, Australia). Trends in the total numbers of private alleles (*A*_p_) reflected the divergence between ocean basins and support very limited inter-basin gene flow, with more than 25% of total SNPs genotyped containing private alleles within each basin; (2,672 and 2,508 for Indian and Pacific Oceans respectively). Within ocean basins, little difference (~2% of total SNPs) was seen among Pacific populations (*A*_p_ range of 188–205), while greater differences (~3–5% total SNPs) were observed among the Abrolhos Islands, both Tanzanian, and Iranian sites (290, 354 and 458 respectively).

**Table 1:** Genetic diversity indices for the *P. margaritifera* populations sampled

Population	n	Source	*N*_*eLD*_ [95% C.I.]	*A*	*Ar* (<5%)	*H*_*o*_ (± SD)	*H*_n.b._ (± SD)	*F*_is_ (p < 0.01)	Av. MLH (± SD)	HL (± SD)	SH (± SD)	IR (± SD)
Australia Abrolhos Island	50	Farm (hatchery)	9.3 [9.3 – 9.4]	1.5649	0.5446	0.0748 (±0.1244)	0.1655 (±0.1924)	0.5542	0.0914 (±0.0115)	0.8592 (±0.0174)	1.0682 (±0.1457)	0.5765 (±0.0516)
Australia GBR	35	Wild	∞ [∞ – ∞]	1.7603	0.3822	0.0762 (±0.0995)	0.2005 (±0.1771)	0.6265	0.0877 (±0.0044)	0.8618 (±0.0073)	1.0189 (±0.0567)	0.5737 (±0.0222)
Cook Islands	45	Farm (wild origin)	1684.7 [1475.1 – 1963.3]	1.6256	0.4984	0.0728 (±0.1092)	0.1722 (±0.1854)	0.5830	0.0868 (±0.0114)	0.8655 (±0.0179)	1.0066 (±0.1398)	0.5888 (±0.0523)
Fiji Islands	61	Farm (wild origin)	232.4 [229.9 – 234.9]	1.7934	0.3895	0.0929 (±0.1151)	0.1991 (±0.1758)	0.5372	0.1030 (±0.0306)	0.8370 (±0.0475)	1.2189 (±0.3905)	0.5050 (±0.1366)
French Polynesia	50	Farm	299.5 [293.4 – 305.9]	1.7208	0.4334	0.0718 (±0.1002)	0.1883 (±0.1814)	0.6236	0.0844 (±0.0091)	0.8687 (±0.0145)	0.9777 (±0.1132)	0.5965 (±0.0416)
Indonesia	48	Wild	1036.3 [972.6 – 1108.9]	1.8067	0.3635	0.0806 (±0.1027)	0.2054 (±0.1739)	0.6121	0.0925 (±0.0137)	0.8543 (±0.0215)	1.0816 (±0.1730)	0.5568 (±0.0633)
Iran	49	Wild	767.8 [693.1 – 860.3]	1.4402	0.7757	0.0371 (±0.0858)	0.1187 (±0.1795)	0.7008	0.0520 (±0.0039)	0.9378 (±0.0056)	0.5830 (±0.0445)	0.8127 (±0.0145)
Papua New Guinea	38	Wild	199.9 [196.3 – 203.8]	1.7632	0.3774	0.0732 (±0.0967)	0.2007 (±0.1769)	0.6410	0.0847 (±0.0034)	0.8661 (±0.0061)	0.9800 (±0.0399)	0.5884 (±0.0152)
Solomon Islands	50	Wild	119.8 [118.9 – 120.8]	1.8001	0.3748	0.0859 (±0.1077)	0.2019 (±0.1739)	0.5790	0.0965 (±0.0211)	0.8471 (±0.0336)	1.1374 (±0.2709)	0.5323 (±0.0964)
Taiwan	24	Wild	∞ [∞ – ∞]	1.7098	0.3830	0.0741 (±0.1035)	0.2021 (±0.1830)	0.6433	0.0859 (±0.0050)	0.8643 (±0.0080)	0.9947 (±0.0648)	0.5864 (±0.0230)
Tanzania (Mafia Island)	35	Wild	∞ [∞ – ∞]	1.4462	0.6369	0.0410 (±0.0878)	0.1296 (±0.1840)	0.6964	0.0553 (±0.0060)	0.9290 (±0.0083)	0.6149 (±0.0715)	0.7910 (±0.0215)
Tanzania (Mtwara)	20	Wild	∞ [∞ – ∞]	1.3934	0.6485	0.0427 (±0.0951)	0.1256 (±0.1871)	0.6795	0.0557 (±0.0083)	0.9285 (±0.0108)	0.6206 (±0.0994)	0.7898 (±0.0299)
Tonga	28	Wild	120.8 [118.7 – 122.8]	1.6995	0.4062	0.0775 (±0.1076)	0.1954 (±0.1828)	0.6119	0.0889 (±0.0104)	0.8594 (±0.0171)	1.0404 (±0.1326)	0.5714 (±0.0493)
Vietnam	47	Wild	681.5 [651.7 – 714.2]	1.8016	0.3587	0.0775 (±0.0994)	0.2060 (±0.1737)	0.6281	0.0892 (±0.0135)	0.8592 (±0.0215)	1.0378 (±0.1706)	0.5723 (±0.0616)

The parameters calculated include the effective population size by the linkage disequilibrium method (*N*_*eLD*_; 95% confidence intervals indicated within brackets), mean number of alleles per locus (*A*), rare allelic richness at (*Ar,* MAF <5%), observed heterozygosity (*H*_o_), average expected heterozygosity corrected for population sample size (*H*_n.b._), inbreeding coefficient (*F*_is_), average individual multi-locus heterozygosity (Av. MLH), homozygosity by locus (HL), standardised heterozygosity (SH) and internal relatedness (IR)

Average observed heterozygosities were significantly lower (*p* < 0.05) than average expected heterozygosities for all populations), and displayed similar variability with the trends observed for *A* and *Ar* values. Pacific Ocean populations displayed generally higher values (*H*_o_: 0.0718–0.0929; *H*_n.b._: 0.1722–0.2060), than did Indian Ocean populations (*H*_o_: 0.0371–0.0748; *H*_n.b._: 0.1187–0.1655). These patterns also extended to individual average multi-locus heterozygosity (MLH) computations, and measurements of standardised heterozygosity (SH). Average MLH was relatively uniform within Pacific Ocean populations, ranging from 0.0844 (French Polynesia) to 0.1030 (Fiji Islands), which was mirrored in the SH results of 0.9777–1.2189 for the same populations respectively. Within Indian Ocean samples, oysters collected from Tanzanian and Iranian sites showed lower values (MLH: 0.0520–0.0557; SH: 0.5830–0.6206), than animals sampled from the Abrolhos Islands (MLH = 0.0914; SH = 1.0682).

Inbreeding coefficient (*F*_is_) values displayed a similar partitioning by region, with values for Pacific Ocean populations ranging from 0.5372 (Fiji Islands) to 0.6433 (Taiwan), while Indian Ocean animals (with the exception of Abrolhos Islands oysters; *F*_is_ = 0.5542), returned higher values from 0.6795 (Tanzania, Mtwara) to 0.7008 (Iran). Very similar patterns were evident in related homozygosity by locus (HL) and internal relatedness (IR) multi-locus metrics (see Table 1). Estimates of effective population size were robust, however, they varied considerably across all sampling locations. Several populations returned infinite *N*_*eLD*_ values, including oysters sampled from the GBR, Taiwan and the two Tanzanian locations. Estimates from Solomon Islands samples were at the low end of the range (119.8; [95% CI = 118.9–120.8]), while Cook Islands individuals produced higher values (1,684.7; [95% CI = 1,475.1–1,963.3]). The lowest estimates were obtained from Abrolhos Islands oysters (9.3; [95% CI = 9.3–9.4]), indicating a possible bottleneck, as these animals were F_1_ hatchery-produced offspring of wild-caught parents.

### Resolution of population structure and migration

Pairwise *F*_st_ estimates (Table 2) were highly significant (*p* < 0.001) for all population comparisons, with the exception of the two Tanzanian sites (0.0007), and PNG with the Solomon Islands (0.0059). A clear separation in population structure between ocean basins is evident, with pairwise estimates between sites all >0.25, ranging from Tanzania, Mtwara and Indonesia (0.2894), to Iran and the Cook Islands (0.4684). Within the Pacific, populations appear to be isolated by geographic separation, e.g. pairwise estimates for the GBR and Solomon Islands (0.0078) indicate greater homogeneity than more distant population pairs, such as the Cook Islands and Taiwan (0.1090). Higher degrees of separation are apparent within Indian Ocean populations, with pairwise estimates between Iran, and Mafia Islands with Mtwara being 0.2444 and 0.2534 respectively. The greatest level of differentiation among Indian Ocean sites was detected between the Abrolhos Islands and Iran (0.4177), with oysters from the Abrolhos Islands demonstrating greater similarity with Pacific populations (Abrolhos Islands and GBR pairwise *F*_st_ = 0.1311).

**Table 2:** Population differentiation estimates for 14 *P. margaritifera* populations sampled

	Australia Abrolhos Islands	Australia GBR	Cook Islands	Fiji Islands	French Polynesia	Indonesia	Iran	Papua New Guinea	Solomon Islands	Taiwan	Tanzania (Mafia Island)	Tanzania (Mtwara)	Tonga	Vietnam
Australia Abrolhos Islands		0.056	0.095	0.068	0.082	0.053	0.264	0.056	0.057	0.053	0.236	0.238	0.069	0.051
Australia GBR	0.1311		0.033	0.009	0.021	0.009	0.256	**0.005**	0.005	0.009	0.234	0.236	0.011	0.008
Cook Islands	0.2173	0.0816		0.023	0.020	0.044	0.306	0.035	0.035	0.044	0.289	0.291	0.027	0.043
Fiji Islands	0.1526	0.0194	0.0537		0.011	0.018	0.273	0.011	0.010	0.017	0.253	0.255	0.006	0.016
French Polynesia	0.1892	0.0490	0.0502	0.0221		0.031	0.292	0.022	0.021	0.030	0.275	0.277	0.014	0.028
Indonesia	0.1209	0.0211	0.1084	0.0459	0.0759		0.243	0.008	0.009	**0.006**	0.217	0.219	0.020	0.006
Iran	0.4177	0.4145	0.4684	0.3903	0.4438	0.3711		0.255	0.257	0.241	0.071	0.074	0.276	0.239
Papua New Guinea	0.1297	0.0079	0.0862	0.0227	0.0532	0.0166	0.40860		0.005	**0.008**	0.232	0.233	0.013	0.007
Solomon Islands	0.1297	0.0071	0.0835	0.0248	0.0499	0.0208	0.38462	**0.0056**		0.008	0.234	0.237	0.012	0.008
Taiwan	0.1196	0.0172	0.1090	0.0375	0.0739	0.0100	0.41411	0.0148	0.0128		0.217	0.218	0.020	**0.006**
Tanzania (Mafia Island)	0.3508	0.3494	0.4185	0.3374	0.3951	0.3038	0.24438	0.3444	0.3236	0.3394		0.013	0.257	0.214
Tanzania (Mtwara)	0.3402	0.3323	0.4043	0.3235	0.3804	0.2894	0.25340	0.3280	0.3084	0.3200	**0.0069**		0.259	0.216
Tonga	0.1607	0.0235	0.0628	0.0099	0.0294	0.0494	0.44413	0.0279	0.0254	0.0443	0.3846	0.3644		0.019
Vietnam	0.1128	0.0185	0.1062	0.0407	0.0732	0.0124	0.37119	0.0161	0.0174	0.0088	0.3043	0.2906	0.0469	

Population pairwise *F*_st_ estimates computed in Arlequin v.3.5.1.3. are shown below the diagonal, while Nei’s (1978) standard genetic distances (*D*_S_) computed in Genetix v.4.05.2 with 10,000 permutations are reported above. All *F*_st_ values were significant at *p* < 0.001 following 1,000 permutations. Non-significant *F*_st_ and *D*_S_ values (*p* > 0.05) are presented in bold type

Pairwise Nei’s standard genetic distances (*D*_S_) described a similar pattern to the pairwise *F*_st_ estimates (Table 2), with the Iranian and two Tanzanian populations displaying marked separation from all other populations (0.214–0.306; *p* < 0.05). Partitioning between these populations however, was less evident, with *D*_S_ = 0.071 and 0.074 respectively (Iran with Mafia Islands and Mtwara). Distances between all Pacific Ocean populations conversely indicated greater homogeneity, ranging from 0.005 (PNG, GBR and Solomon Islands pairwise comparisons), to 0.044 (Cook Islands with Indonesia and Taiwan pairwise comparisons). Oysters collected from the Abrolhos Islands were similarly differentiated, with *D*_S_ = 0.056 when compared to GBR individuals, and up to *D*_S_ = 0.082 with French Polynesian animals.

**Fig. 2:**
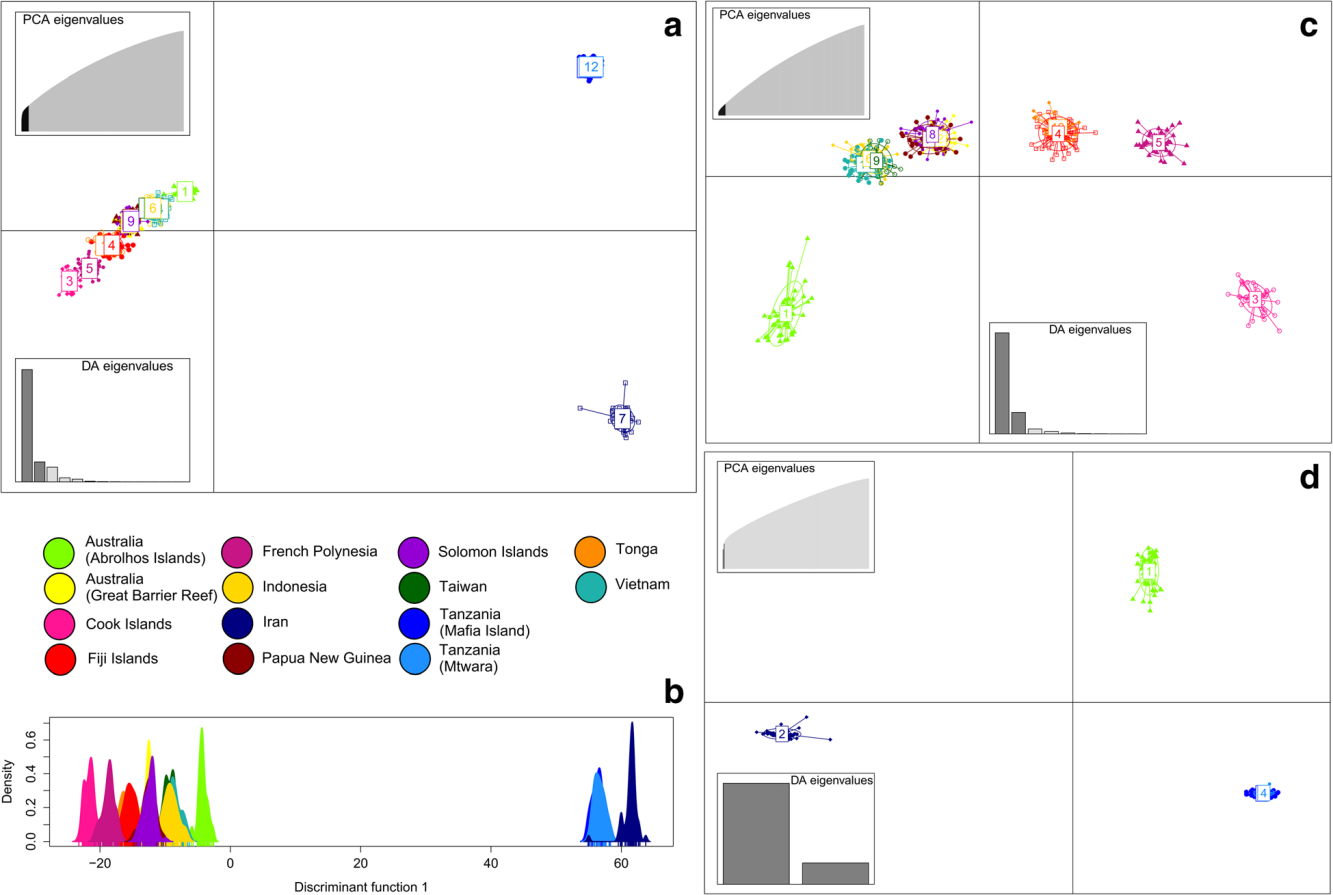
Discriminant Analyses of Principal Components (DAPC) carried out using the *R* package *adegenet* to illustrate broad-scale patterns of population structure. Dots on scatterplots represent individuals, with colours denoting sampling origin and inclusion of 95% inertia ellipses. Scatterplot (**a**) was constructed among all 580 individuals collected from both the Pacific and Indian Ocean sites, while (**b**) is an individual density plot on the first discriminant function for this dataset. Scatterplots (**c**) and (**d**) were constructed on individuals sampled from Pacific Ocean (**c**) and Indian Ocean (**d**) sites only, to clearly identify regional differentiation

Results of the hierarchical AMOVA carried out between Indian vs. Pacific Ocean basins and populations indicated that 18.11% of the variance originated between ocean basins, with the greatest proportions of variance attributed to within-sample variation (45.79%), and between samples within populations (35.74%). Variation between populations within ocean basins was estimated at just 0.36%, indicating that genotypic variability at the individual oyster level accounted for the majority of the observed variation. Mantel tests indicated isolation by distance dispersal patterns both within each ocean basin (R^2^ = 0.939, *p* = 0.041 and R^2^ = 0.464, *p* = 0.000 for Indian and Pacific oceans respectively), as well as for all populations considered together (R^2^ = 0.613, *p* = 0.000), although additional sampling within each region is needed to confirm the strength of these results. Further Mantel tests within the two largest Pacific Ocean population groupings did not detect significant IBD patterns (*p* > 0.05). Visualisation of population structure with a DAPC (α-score optimised to retain 22 PCs), revealed clear differentiation between all Pacific Ocean, and both Tanzanian and Iranian populations (Fig. 2a and b), when all individuals were analysed together. Further DAPC analyses involving separation of populations into their respective ocean basins further clarified the patterns observed. Analysis of all populations from the Pacific Ocean (Fig. 2c) revealed clear partitioning of the French Polynesian and Cook Islands oysters from all other populations, while animals sampled from Fiji and Tonga formed a single cluster. Similarly, individuals collected from PNG, Solomon Islands and the GBR formed a single cohesive group, as did oysters sampled from Indonesia, Taiwan and Vietnam. This pattern of separation was confirmed by testing for the actual number of discrete clusters using the BIC method, which was determined to be k = 8.

**Fig. 3:**
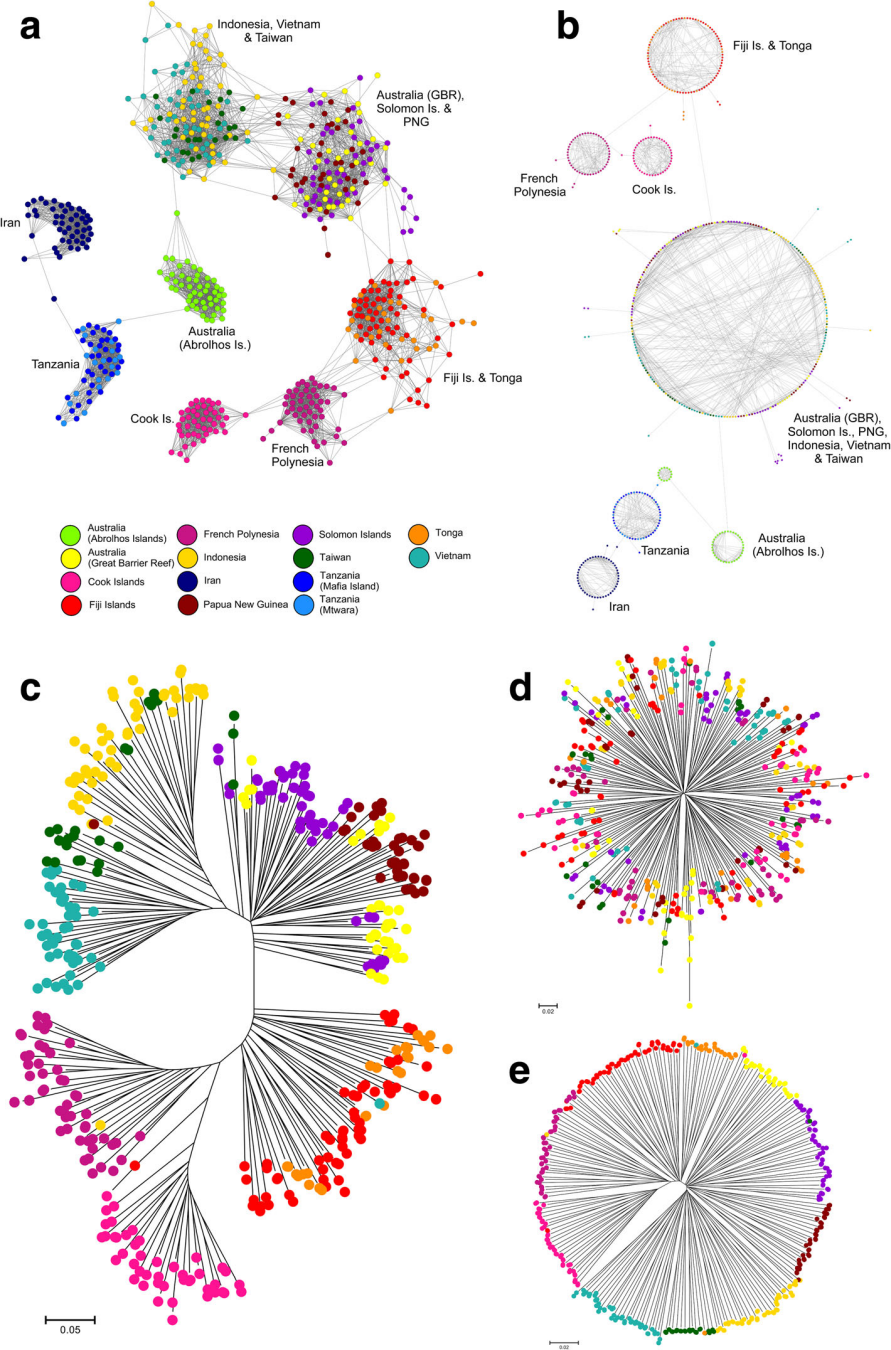
Visualisation of population structure among 580 *P. margaritifera* individuals sampled. Fine-scale population networks constructed using the Netview P v.0.4.2.5 pipeline and selectively-neutral loci are shown in (**a**) organic; k-NN = 40 and (**b**) circular; k-NN = 10 topologies, with each dot representing a single individual. Oysters sampled from the Pacific Ocean had sufficiently low neutral *F*_st_ levels to permit testing for outlier loci, and Neighbour-Joining trees generated based on 1-psa distance matrices for these individuals are shown in (**c**) and (**d**). The tree displayed in (**c**) was drawn using 89 putatively directional outlier loci detected by both Bayescan 2.1 and LOSITAN at an FDR = 0.05, while (**d**) was generated using 37 also jointly-identified putatively balancing loci, at an FDR = 0.05. **e** Shows the arrangement of population structure in these same individuals, but with all loci (9,624 SNPs). The scale bars for (**c**), (**d**) and (**e**) indicate 1-psa genetic distance

Examination of fine-scale population structure using Netview P (Fig. 3a and b) resolved similar patterns of differentiation to the DAPC, but offered greater resolution at the individual oyster level between several population pairs. In particular, when an organic network topology was used (k-NN = 40; Fig. 3a), it highlighted the degree of connectivity between the two broad clusters comprising oysters collected from Indonesia, Vietnam and Taiwan, along with individuals sampled from the GBR, Solomon Islands and PNG respectively. Analysis using a circular network topology (k-NN = 10; Fig. 3b) made this especially clear, as all individuals from these six locations collapsed into a single cluster. Interestingly, oysters collected from the Abrolhos Islands split into two sub-clusters (Fig. 3b), potentially indicating the presence of family groups, given that all individuals were sampled as a hatchery-produced cohort. Similarly, a closer relationship was apparent between French Polynesian, and Fijian-Tongan samples than with Cook Islands individuals, despite the greater geographic distance separating these populations. This may be due to prevailing ocean current patterns, which ensure greater connectivity through directional larval dispersal. Networks constructed at lower and higher k-NN thresholds all showed identical differentiation patterns.

**Fig. 4:**
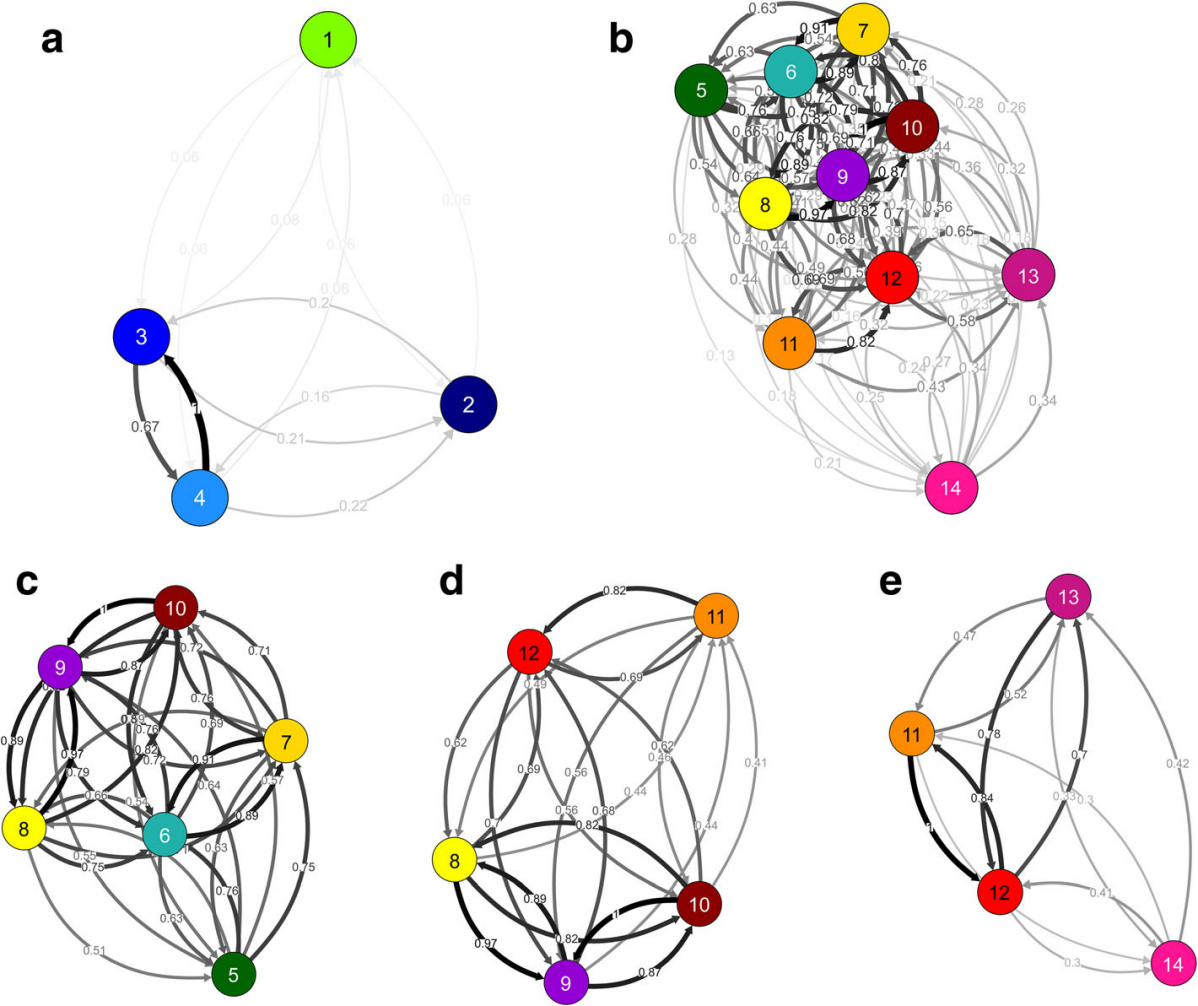
Migration networks for *P. margaritifera* populations generated using the *divMigrate* function in *diveRsity* [72]. Circles represent populations, while arrows indicate the direction and magnitude (arrow edge values) of relative migration levels using Nei’s *G*_st_ method [67, 81]. Darker arrows indicate stronger migration relationships compared to lighter arrows. Separate networks are shown for all Indian Ocean populations (**a**) and all Pacific Ocean populations (**b**) sampled. To better visualise separation between all Pacific Ocean populations, further networks have been generated for population groups located in the Western Pacific (**c**), Western and Central Pacific (**d**) and the Central and Eastern Pacific (**e**). All networks were generated following 1,000 bootstraps and all pairwise relationships are significant (*p* < 0.01). Population colour codes correspond to Figs. 1, 2 and 3, and have been numbered as follows. 1: Australia (Abrolhos Is.), 2: Iran; 3: Tanzania (Mafia Is.), 4: Tanzania (Mtwara), 5: Taiwan, 6: Vietnam, 7: Indonesia, 8: Australia (GBR), 9: Solomon Is., 10: Papua New Guinea, 11: Tonga, 12: Fiji Is., 13: French Polynesia and 14: Cook Is

Assessment of migration patterns and gene flow (Fig. 4) using *divMigrate* networks demonstrated nearly identical patterns of population structure between Indian (Fig. 4a) and Pacific (Fig. 4b) Ocean basins, when compared to the DAPC and Netview P networks. These similarities extended to closer examinations of Pacific Ocean populations by sub-region (Fig. 4c-e). Among Indian Ocean populations, directional migration between both Tanzanian sites was the strongest, but with very little connectivity between these two locations, Iran and the Abrolhos Islands. Connectivity within the Pacific region however, was substantially higher, with only the Cook Islands and French Polynesian populations remaining relatively isolated (Fig. 4b, e). Directional migration between Western Pacific sites (Vietnam, Indonesia, Taiwan, PNG, Solomon Islands and GBR) was found to be the strongest (Fig. 4c, e), followed by connectivity between the Fiji Islands and Tonga (Fig. 4d, e). Despite the geographic proximity of the Cook Islands to the Fiji Islands and Tonga, migration between both these locations and French Polynesia was considerably higher.

### Examination of adaptive variation

*F*_st_ outlier tests discovered between 45 and 137 putatively directional, and 37–216 putatively balancing outlier loci jointly-identified by Bayescan 2.1 and LOSITAN, at six FDR thresholds for Pacific Ocean populations (Additional files 2 and 3). Both platforms failed to detect loci under balancing selection below an FDR = 0.01, and based on verification of loci detected at all FDR thresholds using QQ plots, a final stringent FDR threshold of 0.05 was selected. At this FDR, 89 directional and 37 balancing loci were jointly-identified, and used to construct NJ trees to visualise population structure at loci putatively under selection (Fig. 3c, d and e).

Weak population structure observed at selectively neutral and balancing loci (Fig. 3e and d respectively), correlated well with pairwise *F*_st_ and *D*_S_ comparisons. At directional loci however, clear divergence was evident between populations, which corresponded exactly with the five clusters identified by DAPC and Netview P networks in the Pacific Ocean. To gauge the strength of the selection signal, average Bayescan 2.1 *F*_st_ values among the 89 directional loci were examined, and found to equal 0.1915 (range = 0.1012 to 0.4371). Among the 37 balancing loci, average *F*_st_ = −0.0066 (range = −0.0114 to −0.0031), demonstrating that diffuse population structure (NJ trees Fig. 3e and d), becomes apparent when considering these and selectively neutral loci. These results indicate the likely presence of local adaptation acting on the populations examined, which is likely due to the heterogenous habitats occupied by *P. margaritifera* across the Pacific Ocean.

### Particle dispersal modelling

**Fig. 5:**
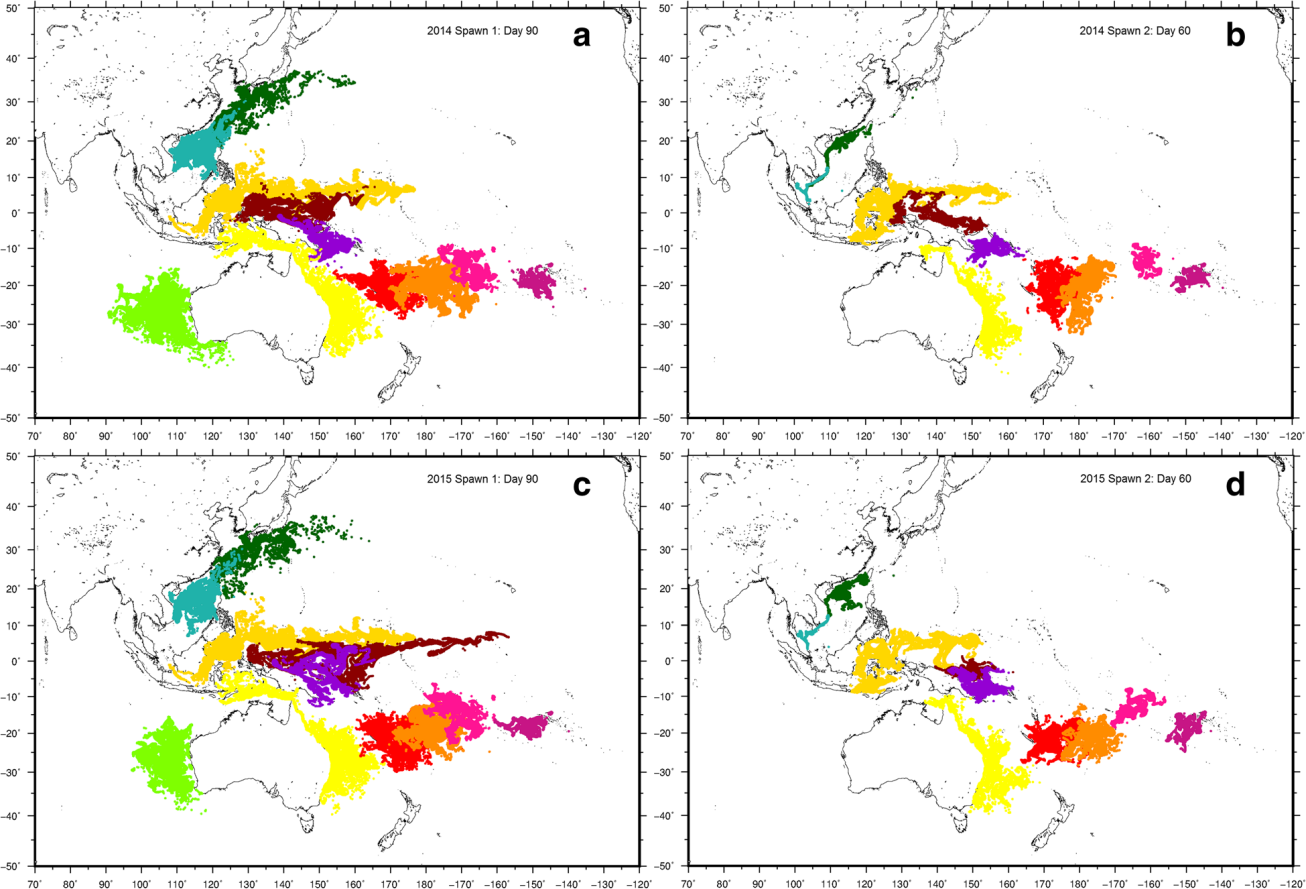
Results of particle dispersal simulation for 11 sampling sites. Particle positions are displayed for the following simulations: spawning season 1 for 2014 (**a**), season 2 for 2014 (**b**), season 1 for 2015 (**c**) and season 2 for 2015 (**d**). All season 1 simulations were run for 90 days, and season 2 simulations over 60 days. Sampling site colour codes correspond with Figs. 1, 2, 3 and 4

Simulations of larval transport revealed a high degree of admixture by surface ocean currents within the Pacific basin over both 2014 and 2015 datasets, (Fig. 5 and see Additional files 4 a, b, c and d for animations of the full dispersal simulations). Interestingly, differences in the direction and extent of dispersal were observed between spawning seasons within either year, than between peak ENSO activity (2014 recorded an El Niño event, which dissipated in 2015). In particular, particles originating in both Taiwan and Vietnam were advected north towards Japan and the Ogasawara Islands archipelago during the first spawning seasons of both 2014 and 2015 (Additional file 1 a, c), while these current patterns reversed during the second spawning seasons, directing particles south across the Vietnamese coastline towards Malaysia (Additional file 1 b, d).

Overall patterns of population structure inferred from DAPC, Netview P and *divMigrate* analyses were highly concordant with simulated dispersal patterns for both ocean basins. At a broad scale, connectivity between the GBR, Solomon Islands, PNG, Indonesia, Vietnam and Taiwan was particularly obvious, together with the Fiji Islands and Tonga. Dispersal patterns for Indian Ocean sampling sites was limited to the Abrolhos Islands, where larval output is likely to spread northwards over much of the Western Australian seaboard (Fig. 5a and c). While providing unprecedented insights into the larval connectivity of *P. margaritifera*, these results should not be interpreted as reflecting actual recruitment over the limits of final particle positions. For example, because larval competency behaviour was not modelled, particles originating from the GBR transported into the South Tasman Sea are unlikely to survive due to unfavourable water temperatures in that region.

## Discussion

This study examined range-wide population genetic structure and connectivity in the black-lip pearl oyster, over its ~18,000 km natural distribution. Assessments of differentiation at both neutral and adaptive markers, together with an independent particle dispersal simulation indicate that the evolutionary and physical processes organising population genetic structure are highly complex. At broad and regional scales, surface ocean currents, geographic distance and habitat geomorphology play important roles in regulating connectivity. At sub-regional and local scales, seascape features such as coral atolls, shoals and straits may impede gene flow, and the presence of environmental heterogeneity result in adaptive differences between populations.

In the Pacific Ocean, our observations do not lend support for a strong CPH model, where *P. margaritifera* is expected to exhibit reduced diversity and increased differentiation towards its range limits. However, this does not imply that CPH trends are absent, as very high levels of gene flow may conceal *C-M* gradients and sampling may not have detected the true range limits. The presence of local adaptation in habitat sub-regions also supports the presence of hetereogenous environments. Conversely in the Indian Ocean, clear divergence between the marginal populations sampled suggests the presence of *C-M* clines cannot be discounted, and requires further investigation at higher sampling densities, with particular attention to central populations. It is apparent that the mechanisms underlying range-wide genetic structure in *P. margaritifera* are quite complex, and require closer examination to better understand the evolutionary, ecological and physical factors at work.

### Basin-wide population structure and connectivity

At a broad scale, *P. margaritifera* populations in the Indian and Pacific Oceans displayed substantial and significant divergence (pairwise *F*_st_ estimates = 0.2894–0.4684, *p* < 0.001). Strong population structure was evident within and between both ocean basins, however, due to the relative isolation of populations between these regions, each is discussed separately.

### Pacific Ocean

Gene flow among Pacific Ocean populations appears to occur at a basin-wide scale, with pairwise *F*_st_ estimates reaching a maximum of 0.1090 (Cook Islands and Taiwan), over a distance of approximately 9,900 km. Despite the high degree of admixture among populations, visualisation of population structure (Figs. 2, 3, and 4) resolved five distinct genetic groups. When dispersal simulation data (Fig. 5 and Additional file 4 a-d) are compared to genetic differentiation patterns, the physical limits of simulated larval dispersal closely match population groupings. This observation suggests that while surface ocean currents permit sufficient gene flow across the Pacific Ocean to ensure populations retain a high degree of connectivity, circulation patterns and IBD may also facilitate regional larval retention, that stabilises population genetic structure. Because even low levels of gene flow [101, 102] are able to prevent population divergence, it is conceivable that standing genetic diversity and structure are maintained by a “founder takes all” density-dependent effect [103], where individuals arriving after an initial colonisation event may be “blocked” by established conspecifics [11, 103].

For the present study, at the geographical limits of the species distribution in the Pacific, decreased differentiation between Taiwan and French Polynesia (*F*_st_ = 0.0739) is evident despite the considerable distance involved (~11,000 km). This observation does not support generalised CPH predictions, and is likely a result of greater connectivity of this population pair through ocean current circulation [8, 104]. This is corroborated by dispersal simulation data (Additional file 4 a and c), and supported by pairwise migration analyses (Fig. 4). Larval competency following an extended pelagic dispersal phase is also expected to play a role in recruitment success or failure, as individuals may have greater fitness as a result of shorter and potentially less stressful larval development [105, 106]. Here, ocean currents may impact recruitment rates by permitting increased larval fitness through reduced transport times, meaning that a population pair separated by greater physical distance may share higher connectivity, compared to a neighbouring population pair where larval plumes are vectored in mutually opposite directions or via circuitous pathways [12, 107].

Another factor influencing population structure and connectivity is habitat geomorphology, which is particularly evident in the Western Pacific, where long-range larval dispersal is restricted by the presence of numerous shoals, straits, islands, reefs and semi-enclosed seas [25]. This is reflected in the segregation of Taiwanese, Vietnamese and Indonesian individuals, from oysters collected in PNG, the Solomon Islands and the GBR (Figs. 2, 3 and 4). Similar patterns have been documented in several highly-dispersive marine taxa, ranging from a diatom [108] and limpet [109], to giant clam [110] and mullet [31].

### Signatures of selection in the Pacific basin

Similarities in the patterns of population structure obtained at loci under directional selection (Fig. 3c-e), to spatial arrangements generated by DAPC and Netview P networks at selectively neutral loci (Figs. 2 and 3a-b), reinforce stock boundaries identified for *P. margaritifera* in the Pacific basin. The seascape of the Pacific region has been shaped by complex geological processes, giving rise to considerable habitat heterogeneity [111, 112]. Given the large extent of the species distribution sampled (>11,000 km), it is feasible that the selective differences observed may originate from distinct habitat sub-regions present within the Pacific basin [27, 113, 114].

For range-wide investigations of genetic structure in broadcast spawning marine species, consideration of adaptive variation can be important for uncovering functional differences between populations that might otherwise go undetected. As an example, adaptive divergence in the Atlantic cod related to temperature and salinity clines across the species distribution was detected by Nielsen et al. [33], but not evident within a restricted portion of its range [115], where environmental differences were predicted to be similar. Similarly, our previous study of *P. margaritifera* in the Fiji Islands failed to detect signatures of selection between and among populations [4]; however, results presented here indicate that detectable selection is evident only at the scale of Fijian and Tongan populations considered together.

In certain situations, adaptive differences in the face of high gene flow are the only discriminating factor through which concise fishery management is possible, by disentangling the effects of selection from demographic history, migration and genetic drift [24, 116, 117]. For example, Nayfa and Zenger [32] detected divergent selection between three Indonesian populations of the silver-lip pearl oyster *P. maxima* over ~2,000 km, where functional differences had manifested themselves in commercial fitness trait differences (namely growth rate and shell size [118]). Because the complex life histories of marine taxa may result in greater vulnerability to pre- and post-settlement selective forces [106, 119], the ability to detect these effects on the genetic composition of populations is critical for informing management for aquaculture, translocation, population supplementation and assisted migration [115, 120–122].

### Indian Ocean

Populations sampled from the Indian Ocean displayed substantial vicariance, with the magnitude of separation between the three distinct genetic groups potentially indicating the presence of distinct ESUs, based on *D*_S_ estimates (Table 2; [123–125]). Work by Ranjbar et al. [126] and Cunha et al. [45], suggest that *Pinctada margaritifera* may in fact be a species complex, with populations in the Persian Gulf comprising a distinct ESU. Restriction of gene flow into the Persian Gulf from the greater Indian Ocean by the Strait of Hormuz likely isolates these individuals, and while the current study provides an initial assessment of basin-wide population differentiation for Indian Ocean *P. margaritifera*, further hierarchical sampling is required to determine regional patterns of evolutionary and contemporary genetic structure.

Particular attention to core populations from the central Indian Ocean (Maldives), Madagascar, Arabian Sea, Bay of Bengal, Andaman Sea and Sumatra may resolve these questions, and potentially ascertain the presence of a genetic break between the Indian and Pacific Oceans. Pairwise *F*_st_ estimates and visualisation of genetic structure between the closest marginal populations from the Western Pacific in the current dataset suggest this is a possibility (see Table 2 and Figs. 2, 3), as similar observations have been recorded for other invertebrate taxa [113, 127–129].

### Patterns across the species’ distribution

The CPH predicts that genetic diversity and connectivity should be highest at the centre of a species’ range and decrease towards the periphery, however, our data indicate the presence of patterns which are substantially more complex than generalised CPH predictions. For Pacific populations, easily discernable *C-M* trends were absent, and may mean that the homogenising influence of basin-wide current circulation patterns disrupts any obvious patterns. However, ocean currents together with isolation by geographic distance are also likely to maintain sub-regional population structure (e.g. Miller et al. [130] for the surf clam *Donax deltoides*).

Sample collection for the current study was organised according to the published theoretical distribution of *P. margaritifera* [35], and therefore it is possible that the true species distribution limit may not have been sampled, if it in fact extends beyond the current known range. If edge effects of decreased genetic diversity and marked differentiation are present, further sampling and analysis at the periphery of the species distribution in the Pacific Ocean may detect them. The levels of divergence between Indian Ocean oysters could reflect edge effects, considering that individuals were sampled from the ocean basin margins, however, as no central populations were able to be sampled, this observation cannot be substantiated. In addition to the CPH, other theoretical models for describing population organisation such as source-sink interactions, and range edge disequilibrium [6] warrant consideration. This is because for many species, range margins are often mobile with expansions and contractions over time, and are the result of numerous biotic and abiotic mechanisms [1, 5, 6].

### Drivers of genetic structure and implications for fishery management

It is evident that the biological and physical processes governing population structure and genetic diversity in *P. margaritifera* are complex. In the Pacific Ocean, our data indicate that ocean currents, seascape features and geographic distances are major influences on population connectivity which both disrupts *C-M* clines, and simultaneously stabilises population structure according to basin sub-regions [27]. Broad-scale habitat geomorphology also plays an important role in differentiating populations, by restricting gene flow and influencing sub-regional natural selection. While our sampling scope in the Indian Ocean was insufficiently dense to determine the existence of *C-M* trends, ocean currents may play a large role in maintaining divergent populations. It is possible that a genetic break between the Indian and Pacific Oceans may exist at the South-East Asian archipelago, and further investigation of these populations could provide answers to this question, as it has for other marine invertebrates [127, 129]. Gauging the importance of oceanic circulation for driving population genetic structure and connectivity for *P. margaritifera* would not have been possible without simulations of larval dispersal, and we suggest that oceanographic and/or ecological modelling data is an indispensable component of range-wide investigations of genetic structure in marine organisms, which possess passively dispersing planktonic larvae [131, 132].

Data presented here do not support *P. m.* var. *typica* and *P. m.* var. *cummingi* as sub-species classifications in the Pacific Ocean, given the level of broad-scale admixture detected and absence of evidence for distinct ESUs. Unfortunately, as Hawaiian populations could not be sampled, no conclusion as to the status of *P. m.* var. *galstoffi* may be drawn. However, given the ability of larvae to disperse across the Pacific basin over the span of several generations, it is possible that Hawaiian populations may not be as divergent as previously thought [133]. Conversely, *P. m.* var. *zanzibarensis* and *P. m.* var. *persica* in the Indian Ocean may constitute distinct ESUs, given their substantial divergence from all other populations, although denser basin-wide sampling is required for verification. A comprehensive range-wide phylogenetic analysis of *P. margaritifera* is also needed to assess how many ESUs may be present, and to determine if the black-lip pearl oyster represents a true species complex. Because there are discernable regional morphological differences within *P. margaritifera*, there may be parallels with the Akoya species complex, which also displays morphological variability, high levels of gene flow and has a similarly extensive Indo-Pacific distribution [35, 50].

## Conclusions

Our findings hold regional fishery management implications for Pacific populations of *P. margaritifera*, with the discovery of five distinct genetic stocks in the region. Given the economic importance of pearl oyster aquaculture for several Pacific Island nations [34, 134], this data provides a benchmark for further evaluation of fine-scale population structure at the level of individual countries and territories, to inform localised fishery management policies. Results presented here are also important for fishery management and aquaculture development in other broadcast spawning marine taxa, as an informed approach for designating stock boundaries relies on robust datasets comprising ecological, evolutionary and physical information.

## Additional files

Additional file 1: Summary of temporal seed inputs for particle dispersal model [97, 135–140]. (DOC 32 kb)
Additional file 2: Numbers of putative directional and balancing *F*_st_ outlier loci discovered in *P. margaritifera*. Data are reported following testing of Pacific Ocean populations at six False Discovery Rate thresholds, using BayeScan 2.1 [82] and LOSITAN [84]. Jointly-identified loci were identified using both outlier detection platforms. (DOC 33 kb)
Additional file 3: Summary of numbers of both putatively balancing and directional SNPs detected. Loci are reported following testing of the entire dataset, to identify selectively-neutral SNPs. (DOC 29 kb)
Additional file 4:
**a.** Animation of particle dispersal model simulation using 2014 HYCOM data for spawning season 1. Particle seed location colour codes for 11 populations are identical to those described in Fig. 1. **b.** Animation of particle dispersal model simulation using 2014 HYCOM data for spawning season 2. Particle seed location colour codes for 10 populations are identical to those described in Fig. 1. **c.** Animation of particle dispersal model simulation using 2015 HYCOM data for spawning season 1. Particle seed location colour codes for 11 populations are identical to those described in Fig. 1. **d.** Animation of particle dispersal model simulation using 2015 HYCOM data for spawning season 2. Particle seed location colour codes for 10 populations are identical to those described in Fig. 1. (ZIP 13273 kb)
Additional file 5: Genotypic data. Genotypes of 580 individuals of *P. margaritifera* at 9,624 selectively neutral genome-wide SNPs are included in a standard STRUCTURE format. (ZIP 2057 kb)
Additional file 6: Genotypic data. Genotypes of 580 individuals of *P. margaritifera* at 10,683 adaptive and selectively neutral genome-wide SNPs are included in a standard STRUCTURE format. (ZIP 2276 kb)

## Abbreviations

AMOVA: Analysis of molecular variance
BIC: Bayesian information criterion
CPH: Core-periphery hypothesis
DAPC: Discriminant analysis of principal components
DArT PL: Diversity arrays technology Ltd
DMSO: Dimethyl sulfoxide, C_2_H_6_OS
DNA: Deoxyribonucleic acid
DVM: Dorsoventral measurement
ENSO: El Niño Southern Oscillation
ESU: Evolutionary significant unit
EtBr: Ethidium bromide, C_21_H_20_BrN_3_
FDR: False discovery rate
GBR: Great barrier reef
gDNA: Genomic deoxyribonucleic acid
HL: Homozygosity by locus
HWE: Hardy-Weinberg equilibrium
HYCOM: Hybrid coordinate ocean model
IBD: Isolation by distance
IR: Internal relatedness
JCU: James Cook University, Australia
k-NN: Number of nearest neighbours k-threshold
LD: Linkage disequilibrium
MAF: Minor allele frequency
MLH: Multi-locus heterozygosity
mtDNA: Mitochondrial deoxyribonucleic acid
MU: Management unit
NCBI: National centre for biotechnology information (U.S.A.)
NJ: Neighbour-joining
PCR: Polymerase chain reaction
PIC: Polymorphic information content
PNG: Papua New Guinea
QQ-plot: Quantile-Quantile plot
RE: Restriction enzyme
SH: Standardised heterozygosity
SNP: Single nucleotide polymorphism
