# Supplementary material for: Swept away: ocean currents and seascape features influence genetic structure across the 18,000 Km Indo-Pacific distribution of a marine invertebrate, the black-lip pearl oyster Pinctada margaritifera
Source: BMC Genomics. 2017 Jan 10;18:66. doi: 10.1186/s12864-016-3410-y (PMC5225542; doi:10.1186/s12864-016-3410-y)
Supplement: Additional file 1: — Summary of temporal seed inputs for particle dispersal model [97, 135–140]. (DOC 32 kb) [file 12864_2016_3410_MOESM1_ESM.doc]

**Additional file 1. Summary of temporal seed inputs for particle dispersal model.**

| **Sampling site** | **Particle seed #1 start date (Spawning peak 1)** | **Particle seed #2 start date (Spawning peak 2)** | **References** | **Comment** |
| --- | --- | --- | --- | --- |
| Australia  Abrolhos Islands. | October 1 | None | [97, 135–137] |  |
| Taiwan | June 1 | None | [138] |  |
| Vietnam | June 1 | November 1 | [138] |  |
| Indonesia | June 1 | November 1 | [97] | Inferred from data for spawning behaviour in documented from Vietnam and Indonesia. |
| Papua New Guinea | July 1 | November 1 | [97] | Inferred from data for spawning behaviour documented from the Great Barrier Reef. |
| Solomon Islands | July 1 | November 1 | [97] | Inferred from data for spawning behaviour documented from the Great Barrier Reef. |
| Australia  Great Barrier Reef | July | November 1 | [135] |  |
| Fiji Islands | March 1 | November 1 | [48, 139] |  |
| Tonga | March 1 | November 1 | [97] | Inferred from data for spawning behaviour documented from the Fiji Islands. |
| Cook Islands | May 1 | November 1 | [97] | Inferred from data for spawning behaviour documented from French Polynesia. |
| French Polynesia | May 1 | November 1 | [140] |  |
