# Supplementary material for: Swept away: ocean currents and seascape features influence genetic structure across the 18,000 Km Indo-Pacific distribution of a marine invertebrate, the black-lip pearl oyster Pinctada margaritifera
Source: BMC Genomics. 2017 Jan 10;18:66. doi: 10.1186/s12864-016-3410-y (PMC5225542; doi:10.1186/s12864-016-3410-y)
Supplement: Additional file 2: — Numbers of putative directional and balancing F st outlier loci discovered in P. margaritifera. Data are reported following testing of Pacific Ocean populations at six False Discovery Rate thresholds, using BayeScan 2.1 [82] and LOSITAN [84]. Jointly-identified loci were identified using both outlier detection platforms. (DOC 33 kb) [file 12864_2016_3410_MOESM2_ESM.doc]

**Additional file 2. Numbers of putative directional and balancing *F*_st_ outlier loci discovered in *P. margaritifera***. Data are reported following testing of Pacific Ocean populations at six False Discovery Rate thresholds, using BayeScan 2.1 [82] and LOSITAN [84]. Jointly-identified loci were identified using both outlier detection platforms.

| **FDR level** | **Bayescan 2.1 total outliers** | **Bayescan 2.1**  **directional** | **Bayescan 2.1 balancing** | **LOSITAN directional** | **LOSITAN**  **balancing** | **Jointly-identified**  **directional** | **Jointly-identified**  **balancing** |
| --- | --- | --- | --- | --- | --- | --- | --- |
| 0.001 | 310 | 310 | 0 | 513 | 434 | 87 | 0 |
| 0.005 | 396 | 396 | 0 | 254 | 116 | 55 | 0 |
| 0.01 | 429 | 429 | 0 | 241 | 124 | 45 | 0 |
| 0.05 | 571 | 517 | 54 | 418 | 211 | 89 | 37 |
| 0.10 | 700 | 585 | 115 | 518 | 405 | 112 | 90 |
| 0.20 | 950 | 701 | 249 | 592 | 1215 | 137 | 216 |
