# Supplementary material for: Swept away: ocean currents and seascape features influence genetic structure across the 18,000 Km Indo-Pacific distribution of a marine invertebrate, the black-lip pearl oyster Pinctada margaritifera
Source: BMC Genomics. 2017 Jan 10;18:66. doi: 10.1186/s12864-016-3410-y (PMC5225542; doi:10.1186/s12864-016-3410-y)
Supplement: Additional file 3: — Summary of numbers of both putatively balancing and directional SNPs detected. Loci are reported following testing of the entire dataset, to identify selectively-neutral SNPs. (DOC 29 kb) [file 12864_2016_3410_MOESM3_ESM.doc]

**Additional file 3. Summary of numbers of both putatively balancing and** **directional SNPs detected.** Loci are reported following testing of the entire dataset, to identify selectively-neutral SNPs.

| **FDR**  **threshold** | **Number of outlier SNPs detected by LOSITAN** | **Number of outlier SNPs detected by Bayescan 2.1** | **Jointly identified**  **SNPs** |
| --- | --- | --- | --- |
| 0.001 | 6403 | 620 | 423 |
| 0.005 | 4397 | 901 | 487 |
| 0.01 | 4843 | 1059 | 621 |
| 0.05 | 5778 | 1605 | 1085 |
| 0.1 | 6480 | 1956 | 1392 |
| 0.2 | 7285 | 2498 | 1871 |

For Additional files 4a-d, please note that these.GIF files need to be opened in a web browser to display correctly.
