# Supplementary figures and images for: Swept away: ocean currents and seascape features influence genetic structure across the 18,000 Km Indo-Pacific distribution of a marine invertebrate, the black-lip pearl oyster Pinctada margaritifera
Source: BMC Genomics. 2017 Jan 10;18:66. doi: 10.1186/s12864-016-3410-y (PMC5225542; doi:10.1186/s12864-016-3410-y)

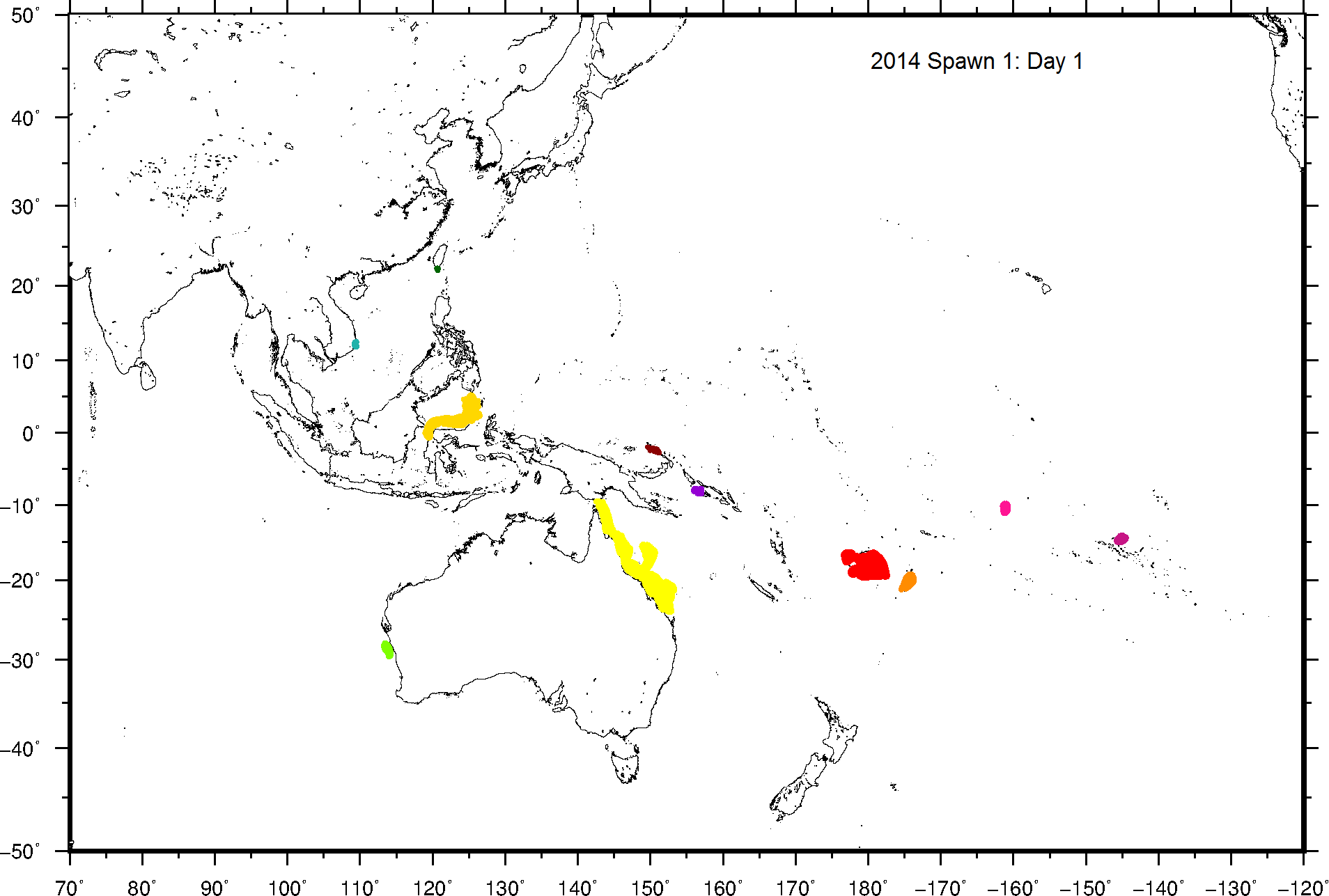

Supplement: Additional file 4: — a. Animation of particle dispersal model simulation using 2014 HYCOM data for spawning season 1. Particle seed location colour codes for 11 populations are identical to those described in Fig. 1. b. Animation of particle dispersal model simulation using 2014 HYCOM data for spawning season 2. Particle seed location colour codes for 10 populations are identical to those described in Fig. 1. c. Animation of particle dispersal model simulation using 2015 HYCOM data for spawning season 1. Particle seed location colour codes for 11 populations are identical to those described in Fig. 1. d. Animation of particle dispersal model simulation using 2015 HYCOM data for spawning season 2. Particle seed location colour codes for 10 populations are identical to those described in Fig. 1. (ZIP 13273 kb) [file 12864_2016_3410_MOESM4_ESM.zip › Additional_File_4a_2014_SPAWN_1.gif]

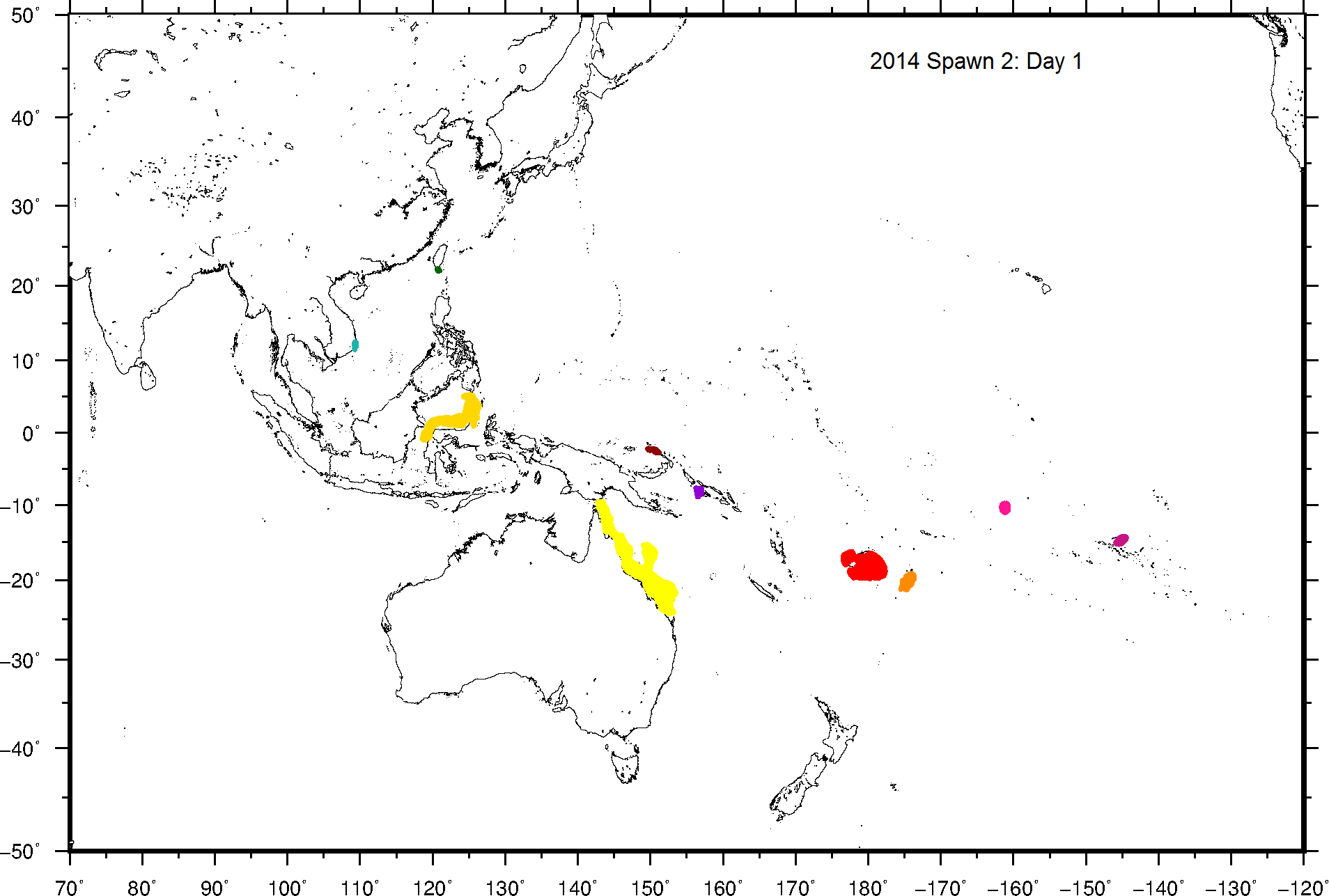

Supplement: Additional file 4: — a. Animation of particle dispersal model simulation using 2014 HYCOM data for spawning season 1. Particle seed location colour codes for 11 populations are identical to those described in Fig. 1. b. Animation of particle dispersal model simulation using 2014 HYCOM data for spawning season 2. Particle seed location colour codes for 10 populations are identical to those described in Fig. 1. c. Animation of particle dispersal model simulation using 2015 HYCOM data for spawning season 1. Particle seed location colour codes for 11 populations are identical to those described in Fig. 1. d. Animation of particle dispersal model simulation using 2015 HYCOM data for spawning season 2. Particle seed location colour codes for 10 populations are identical to those described in Fig. 1. (ZIP 13273 kb) [file 12864_2016_3410_MOESM4_ESM.zip › Additional_File_4b_2014_SPAWN_2.gif]

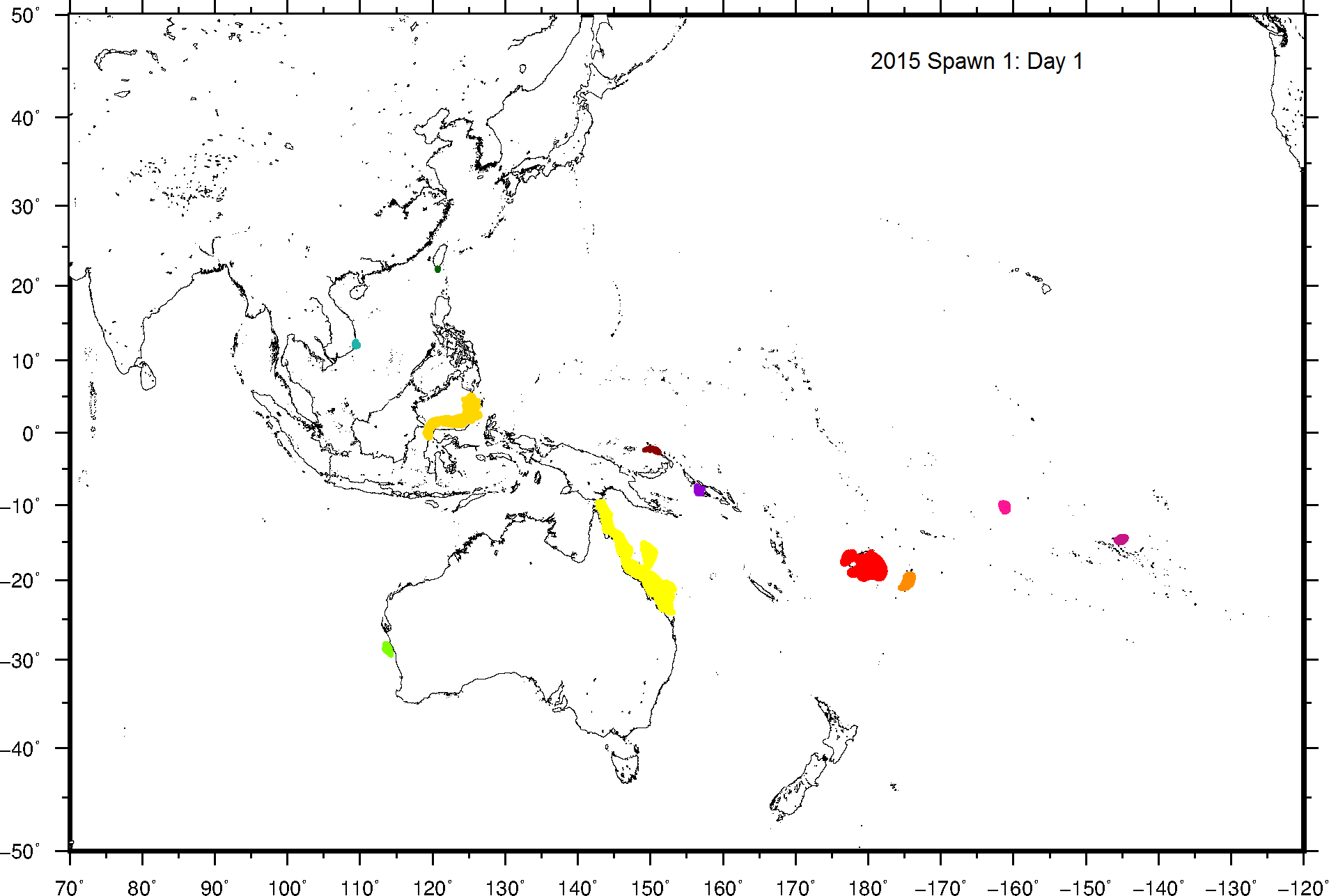

Supplement: Additional file 4: — a. Animation of particle dispersal model simulation using 2014 HYCOM data for spawning season 1. Particle seed location colour codes for 11 populations are identical to those described in Fig. 1. b. Animation of particle dispersal model simulation using 2014 HYCOM data for spawning season 2. Particle seed location colour codes for 10 populations are identical to those described in Fig. 1. c. Animation of particle dispersal model simulation using 2015 HYCOM data for spawning season 1. Particle seed location colour codes for 11 populations are identical to those described in Fig. 1. d. Animation of particle dispersal model simulation using 2015 HYCOM data for spawning season 2. Particle seed location colour codes for 10 populations are identical to those described in Fig. 1. (ZIP 13273 kb) [file 12864_2016_3410_MOESM4_ESM.zip › Additional_File_4c_2015_SPAWN_1.gif]

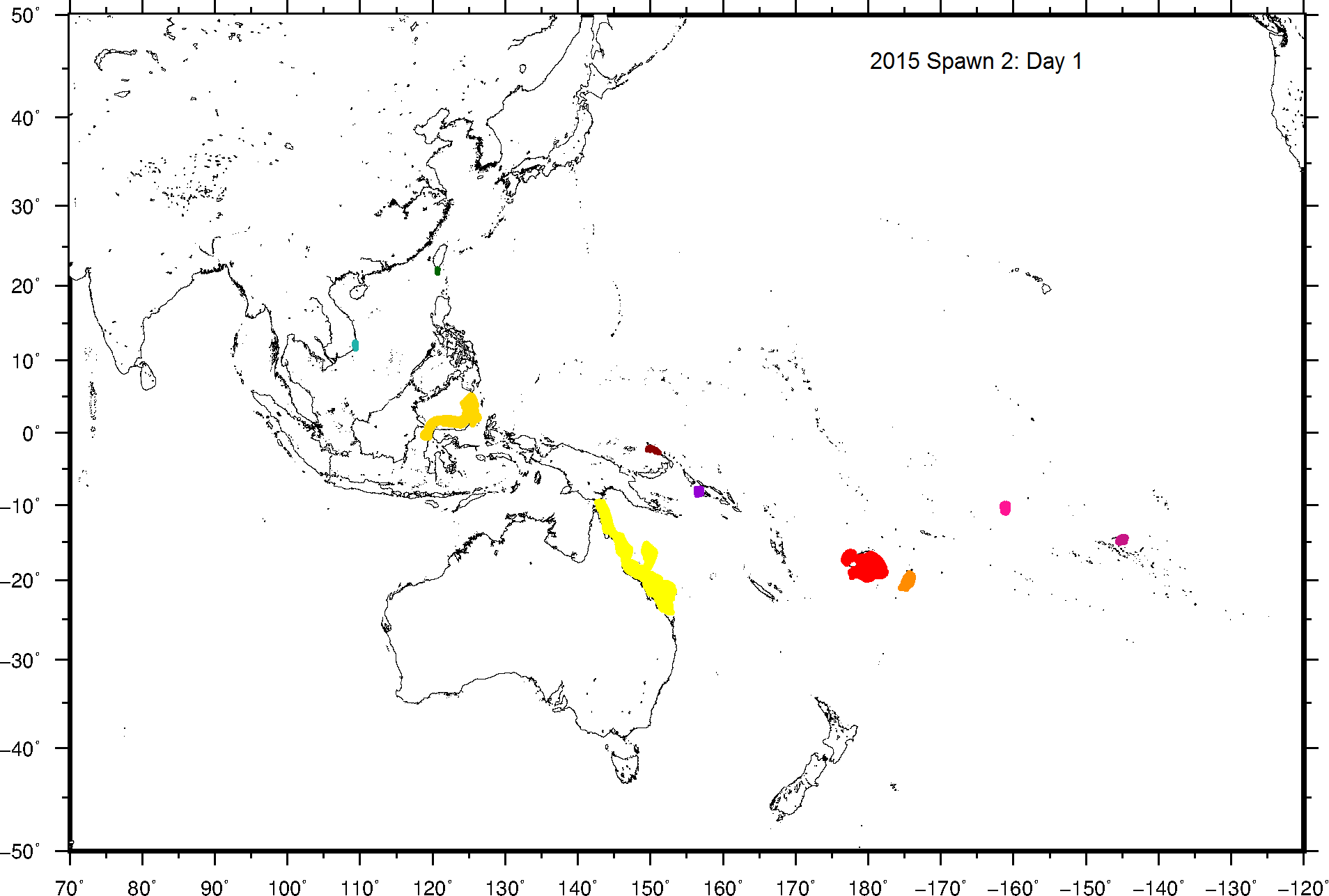

Supplement: Additional file 4: — a. Animation of particle dispersal model simulation using 2014 HYCOM data for spawning season 1. Particle seed location colour codes for 11 populations are identical to those described in Fig. 1. b. Animation of particle dispersal model simulation using 2014 HYCOM data for spawning season 2. Particle seed location colour codes for 10 populations are identical to those described in Fig. 1. c. Animation of particle dispersal model simulation using 2015 HYCOM data for spawning season 1. Particle seed location colour codes for 11 populations are identical to those described in Fig. 1. d. Animation of particle dispersal model simulation using 2015 HYCOM data for spawning season 2. Particle seed location colour codes for 10 populations are identical to those described in Fig. 1. (ZIP 13273 kb) [file 12864_2016_3410_MOESM4_ESM.zip › Additional_File_4d_2015_SPAWN_2.gif]
